# Myoglobin-Catalyzed Azide Reduction Proceeds via an Anionic Metal Amide Intermediate

**DOI:** 10.1021/jacs.3c09279

**Published:** 2024-01-09

**Authors:** Matthias Tinzl, Johannes V. Diedrich, Peer R. E. Mittl, Martin Clémancey, Markus Reiher, Jonny Proppe, Jean-Marc Latour, Donald Hilvert

**Affiliations:** † Laboratory of Organic Chemistry, ETH Zürich, 8093 Zürich, Switzerland; ‡ Institute of Physical and Theoretical Chemistry, TU Braunschweig, 38106 Braunschweig, Germany; § Department of Biochemistry, University of Zürich, 8057 Zürich, Switzerland; ∥ Université Grenoble AlpesCNRS, CEA, IRIG, Laboratoire de Chimie et Biologie des Métaux, 17 Rue des Martyrs, Grenoble F-38054 Cedex, France; ⊥ Institute for Molecular Physical Science, ETH Zürich, 8093 Zürich, Switzerland

## Abstract

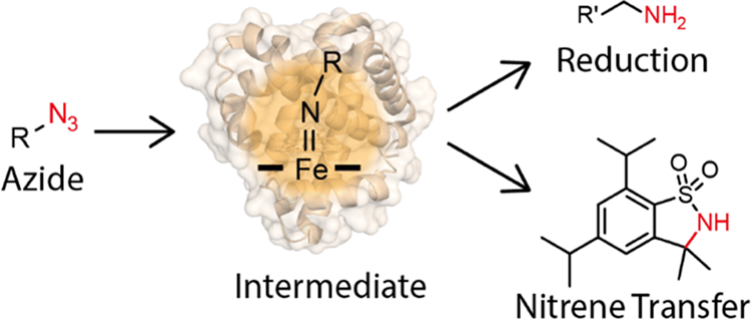

Nitrene transfer reactions catalyzed by heme proteins have broad potential for the stereoselective formation of carbon–nitrogen bonds. However, competition between productive nitrene transfer and the undesirable reduction of nitrene precursors limits the broad implementation of such biocatalytic methods. Here, we investigated the reduction of azides by the model heme protein myoglobin to gain mechanistic insights into the factors that control the fate of key reaction intermediates. In this system, the reaction proceeds via a proposed nitrene intermediate that is rapidly reduced and protonated to give a reactive ferrous amide species, which we characterized by UV/vis and Mössbauer spectroscopies, quantum mechanical calculations, and X-ray crystallography. Rate-limiting protonation of the ferrous amide to produce the corresponding amine is the final step in the catalytic cycle. These findings contribute to our understanding of the heme protein-catalyzed reduction of azides and provide a guide for future enzyme engineering campaigns to create more efficient nitrene transferases. Moreover, harnessing the reduction reaction in a chemoenzymatic cascade provided a potentially practical route to substituted pyrroles.

## Introduction

Biocatalytic nitrene transfer reactions are an attractive option for forming C–N bonds due to their potentially high stereoselectivity and environmentally benign conditions. The special properties of the iron porphyrin cofactor make heme proteins particularly well-suited catalysts for such reactions. For example, cytochrome P450s and cytochrome *c* have been successfully employed to catalyze a wide range of nitrene transfers, including the intramolecular cyclization of arylsulfonyl azides and carbonazidates,^1−3^ aziridinations,^4^ sulfimidations,^5^ aminohydroxylations,^6^ and benzylic and allylic C–H aminations.^7^ However, a major limitation of many of these transformations is the competing reduction of the nitrene precursors to give the corresponding amines. A thorough understanding of the factors that favor reduction over transfer could potentially be leveraged to minimize or even prevent this undesirable side reaction.

To investigate heme-dependent azide reduction, we chose the oxygen storage protein myoglobin as a model heme system. Apart from its native function, myoglobin is remarkably versatile as a catalyst for carbene transfer reactions^8−12^ and additionally exhibits promiscuous peroxidase activity^13−15^—two reaction types that are mechanistically related to nitrene transfer.^16^ Although myoglobin variants can be highly active carbene transferases and peroxidases, the protein is known to catalyze only intramolecular nitrene transfer reactions, such as the cyclization of arylsulfonyl azides.^17^ Even in this case, substrate reduction competes with cyclization. Myoglobin thus represents an ideal scaffold for investigating the reaction of heme proteins with azides.

In this study, we have applied NMR, UV/vis, and Mössbauer spectroscopies as well as X-ray crystallography to gain mechanistic insights into the heme-catalyzed reduction of azides. Identification of a reactive, anionic S = 0 ferrous amide intermediate, the first reactive metal amide to be characterized in a protein scaffold, is a key finding. Furthermore, we show that the myoglobin-catalyzed reduction of azides can be exploited in a chemoenzymatic cascade for the synthesis of substituted pyrroles, demonstrating that this reaction is not just an undesirable side activity but also has potential synthetic value.

## Results

### Reactivity of Myoglobin with Azides and Design of a Chemoenzymatic Cascade

**Figure 1:**
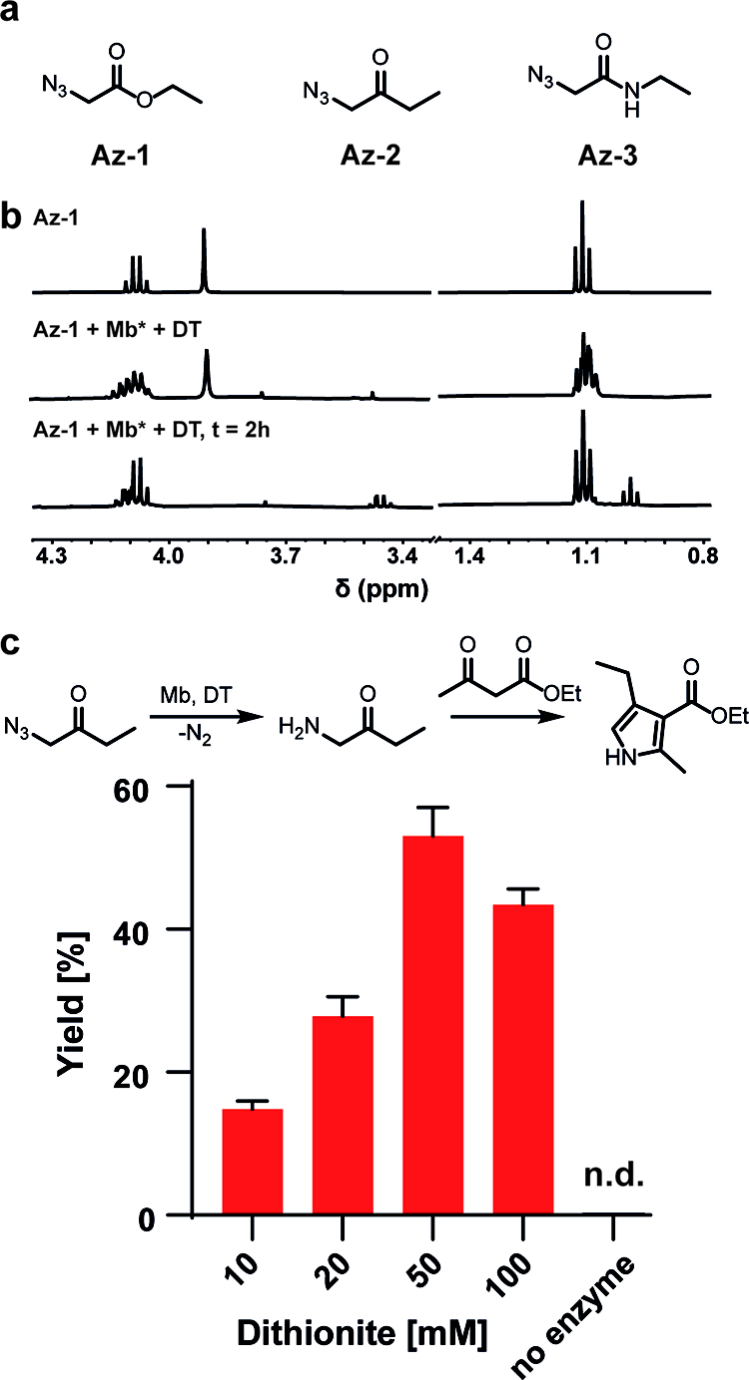
(a) Azides **Az-1**, **Az-2**, and **Az-3** were tested for their reactivity with myoglobin. (b) NMR time course of the reaction of Mb* with **Az-1**. Reaction conditions: 50 μM enzyme, 10 mM azide **Az-1** (added from a 400 mM stock in *d*_6_-DMSO), 10 mM dithionite, in D_2_O under a nitrogen atmosphere. (c) Chemoenzymatic reaction cascade for pyrrole synthesis. After the enzyme-catalyzed reduction of **Az-2**, Knorr pyrrole synthesis occurs spontaneously. Reaction conditions: 20 μM enzyme, 20 mM **Az-2**, 200 mM ethyl acetoacetate, 10–100 mM dithionite, stirring under a nitrogen atmosphere for 18 h; nd: not detected. For the no enzyme control, 50 mM dithionite was used.

For this study, we chose a variant of sperm whale myoglobin that contains two active site mutations (H64V and V68A), which create additional space in the distal binding pocket and are known to boost carbene transferase activity.^8^ We call this protein Mb*. Since Mb* readily reacts with ethyl diazoacetate (EDA),^8−12^ we synthesized a panel of primary azides that are structural analogues of EDA (Figure 1a). When ethyl 2-azidoacetate (**Az-1**), 1-azidobutan-2-one (**Az-2**), and ethyl 2-azidoacetamide (**Az-3**) were incubated with Mb* under a nitrogen atmosphere, no reaction occurred. However, upon addition of the reductant sodium dithionite, the immediate evolution of nitrogen gas was observed for **Az-1** and, to a lesser extent, for **Az-2**. In contrast, **Az-3** did not visibly react with the enzyme under these conditions. In no case was gas evolution observed when the azides were treated with dithionite in the absence of an enzyme.

To gain a better understanding of these findings, we monitored the reactions by NMR. The NMR spectrum of pure **Az-1** consists of a quadruplet with a chemical shift of δ 4.1 ppm (two hydrogens), a singlet at 3.9 ppm (two hydrogens), and a triplet at 1.1 ppm (three hydrogens) (Figure 1b). Upon addition of Mb* and dithionite, the triplet and quadruplet were immediately converted to complex multiplets, suggesting the formation of new species. The singlet also broadened and completely disappeared after 2 h, indicative of fast proton exchange with the D_2_O solvent. In addition, free ethanol was detected (triplet at 0.9 ppm and quadruplet at 3.4 ppm), indicating some hydrolysis. In control experiments in which **Az-1** was incubated only with dithionite, we observed some ethanol formation via ester hydrolysis, but the spectrum was otherwise unchanged. The hydrolytic reaction is likely an acid-catalyzed process associated with the decomposition of dithionite in water (S_2_O_4_^2–^ + 2H_2_O → 2HSO_3_^–^ + 2H^+^ + 2e^–^)^18^ and independent of the protein. Analogous NMR experiments carried out with **Az-2** produced comparable results, but the NMR spectrum of **Az-3** incubated with Mb* and dithionite did not change over 2 h. These findings demonstrate that reactivity strongly depends on the identity of the azide (**Az-1** > **Az-2** ≫ **Az-3**). The lower conformational flexibility of the amide functionality of **Az-3** likely hinders its reaction with the protein-bound heme cofactor.

Reactions of ferrous Mb* with **Az-1** and **Az-2** gave the corresponding free amines as the main products. In the case of **Az-2**, a compound with a molecular weight of 136 Da was also detected and was assigned as 2,5-diethyl pyrazine. The identity of this product was confirmed by comparison with an authentic standard. Pyrazine formation likely proceeds by the reduction of **Az-2** to the amine, followed by dimerization and aromatization.

Intrigued by the latter result, we designed a chemoenzymatic cascade involving enzyme-catalyzed azide reduction of ketone **Az-2** as a first step, followed by Knorr pyrrole synthesis by the reaction of the amine with ethyl acetoacetate. Yields strongly depended on the concentration of dithionite (Figure 1c). Using optimized conditions, yields up to 55% were obtained, which compares favorably with a previously reported transaminase-based biocatalytic method.^19^ In the absence of protein, no product was detected (Figure 1c). Utilization of the cascade is preferred to a one-step reaction of amine and ethyl acetoacetate because in situ amine generation minimizes competing pyrazine formation. An additional advantage of the myoglobin-based cascade is that it can be run at neutral pH without accumulation of undesirable side products, whereas the transaminase system requires pH 5 to suppress pyrazine formation.^19^

**Figure 2:**
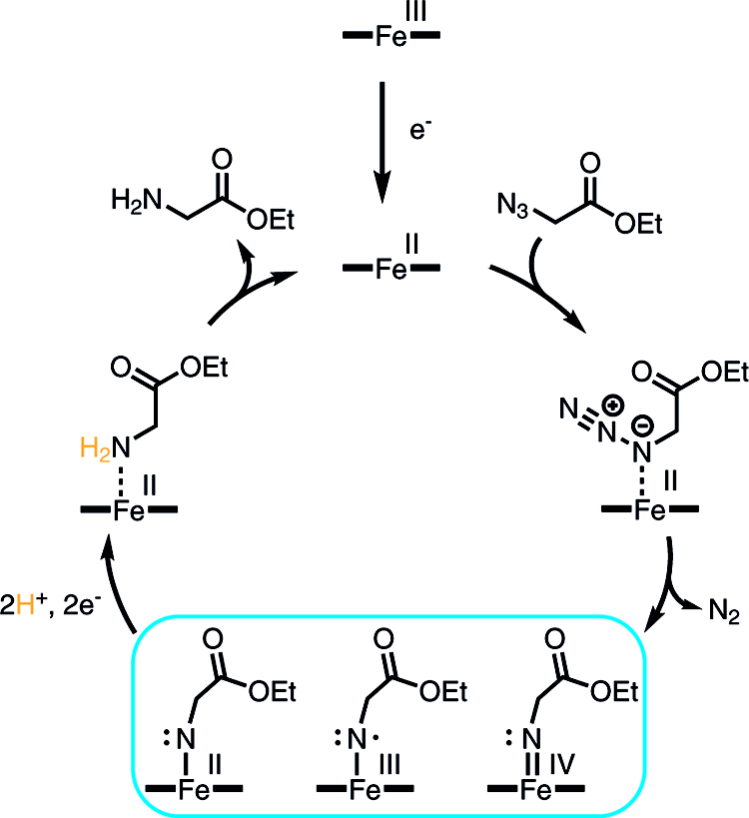
Catalytic cycle for the reduction of azides to amines.

Reduction of **Az-1** and **Az-2** by Mb* in the presence of dithionite likely follows the general mechanism suggested for azide reduction,^1,3,4,20,21^ which is shown in Figure 2. In the first step, the ferric heme cofactor is reduced by dithionite, followed by reaction of the resulting ferrous heme with the azide to produce a nitrene intermediate, which can be formulated as an Fe(IV), an Fe(III) species, or an Fe(II) species (Figure 2). Then, two electrons and two protons have to be transferred to the nitrene to produce the amine and regenerate the ferrous heme catalyst. However, neither the exact sequence of electron and proton transfers nor the electronic properties of the intermediates are known.

### Investigation of the Reduction Reaction by UV/vis Spectroscopy

**Figure 3:**
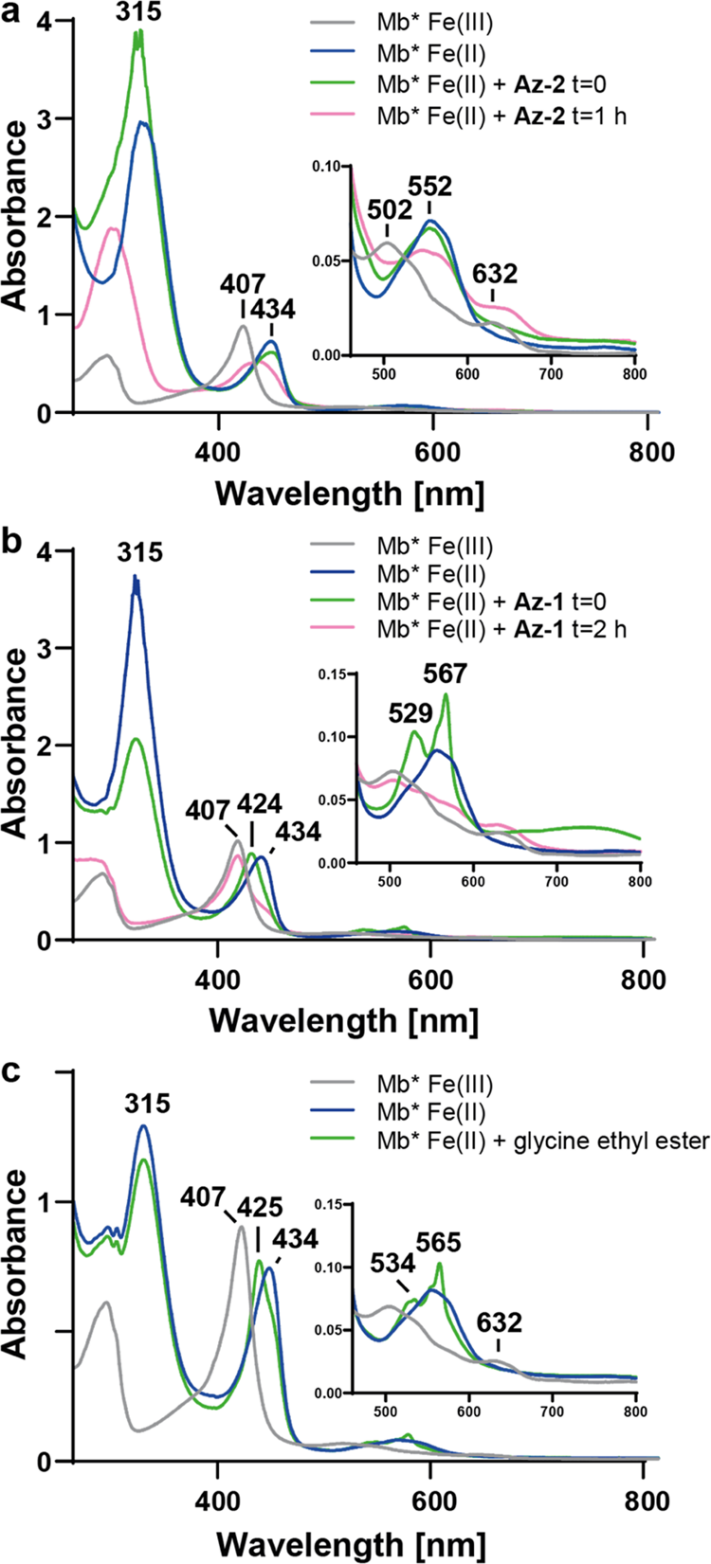
Monitoring reactions of myoglobin by UV/vis. UV/vis spectra were collected upon incubation (a) of Mb* and azidobutanone (**Az-2**), (b) Mb* and ethyl azidoacetate (**Az-1**), or (c) Mb* and glycine ethyl ester. All variants were reduced with dithionite (1 mM) before azide or glycine ethyl ester was added.

Complementing the NMR experiments, we monitored the reaction of Mb* with azides by UV/vis spectroscopy. As expected, in the absence of dithionite, no spectral change was observed during a 1 h incubation of ferric Mb* with **Az-1** or **Az-2**. In the presence of dithionite, Mb*Fe(III) was reduced to ferrous Mb*Fe(II), which is characterized by a Soret band at 434 nm and a Q-band at 552 nm (Figure 3, blue). Upon addition of **Az-2**, the Soret peak broadened and shifted to around 425 nm over the course of 1 h (Figure 3a, pink); a new maximum around 550 nm and a plateau at 630 nm also appeared in the Q-band region. While these changes are indicative of a reaction at the iron center, the broad Soret peak suggests that a mixture of species is probably present in solution, precluding more detailed conclusions.

When the same experiment was carried out with azide **Az-1**, the addition of substrate to ferrous Mb*Fe(II) induced immediate spectral changes (Figure 3b, green). The sharp features of the resulting spectrum suggest that only a single species was produced, which we call Mb*Int. Stopped flow measurements confirmed that Mb*Int is formed in less than 1 s (Figure S1). This species is characterized by a single Soret peak at 424 nm, two sharp features at 529 and 567 nm, and a broad band between 680 and 800 nm (Figure 3b, green). Although this spectrum qualitatively resembles the UV/vis signature of oxygen-bound myoglobin (Mb-O_2_), we ruled out this possibility for two reasons. First, the experiments were performed under a nitrogen atmosphere with an excess of dithionite, and although the **Az-1** solution was not degassed before addition to the cuvette, dithionite rapidly quenches introduced oxygen. Second, the maxima observed in the Q-band region of Mb*Int do not match the characteristic maxima at 541 and 581 nm reported for Mb-O_2_.^22,23^ Nevertheless, we prepared Mb*-O_2_ ourselves for direct comparison with Mb*Int by first reducing Mb* with dithionite and brief bubbling of pure oxygen through the solution before recording the spectrum. Despite rapid autoxidation, we were able to confirm that this species has maxima at 540 and 580 nm (Figure S2), in close agreement with the literature values. We therefore conclude that Mb*Int and Mb*-O_2_ are similar but different species.

The rapid consumption of dithionite in the reaction with **Az-1**, indicated by a progressive decrease in absorbance at 315 nm, suggests that Mb*Int may also be converted to metmyoglobin (Mb*Fe(III)) but is quickly regenerated as long as excess **Az-1** and dithionite are available. This hypothesis was confirmed by running the reaction at high **Az-1**/dithionite ratios (20:1 or greater). When all of the dithionite was consumed, Mb* was recovered in the ferric Fe(III) state (Figure 3b, pink).

To rule out the possibility that Mb*Int is a simple complex of reduced **Az-1** coordinated to the heme, we mixed ferrous Mb* with glycine ethyl ester (GlyOEt). The resulting UV/vis spectrum is characterized by a broad Soret band at 425 nm and Q-bands at 534 and 565 nm (Figure 3c, green). The broad shoulder on the Soret band clearly indicates a mixture of unliganded ferrous Mb* and the GlyOEt complex, suggesting that the product binds the reduced heme cofactor only weakly. Importantly, the Mb*GlyOEt complex does not exhibit a band between 680 and 800 nm, in contrast to Mb*Int. We conclude that the reaction of ferrous Mb* with **Az-1** yields a novel species that is continuously regenerated over a prolonged period in the presence of sufficient **Az-1** and dithionite. We hypothesize that it is generated by the rapid reduction and protonation of a transiently formed nitrene species (Figure 2).

### Capture of the Reactive Intermediate In Crystallo

**Figure 4:**
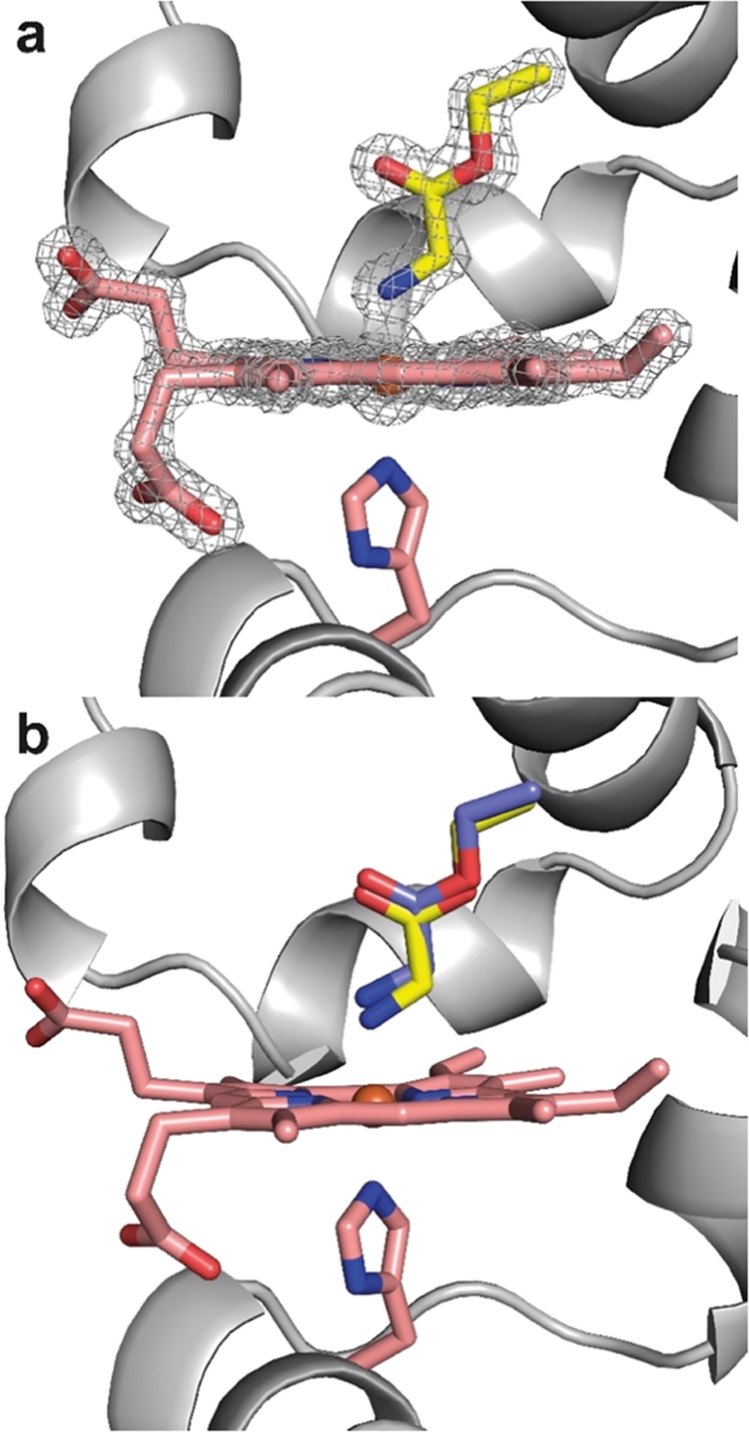
Crystal structures of Mb*. (a) Structure of Mb* soaked with azide **Az-1** (yellow). The *F*_o_ – *F*_c_ omit map (gray mesh) was contoured at 3σ. (b) Overlay of Mb*Int (yellow) and Mb* complexed with glycine ethyl ester (purple). Carbon, oxygen, and nitrogen atoms are shown in pink/yellow/purple, red, and blue, respectively. Iron is shown as an orange sphere.

Encouraged by the spectroscopic results, we attempted to characterize Mb*Int crystallographically. Mb* crystals, which were grown as previously described,^12,14^ were first reduced anaerobically in a Schlenk tube using dithionite; the azide was then added, and the resulting slurry was incubated anaerobically. At different time points, crystals were removed from the Schlenk tube by using a syringe. Single crystals were captured with a loop, dipped into a previously degassed cryoprotectant, and cryo-cooled. Diffraction data were collected, and the structures were solved by molecular replacement (Figure 4, Table S1). The resolution depended on the duration of incubation with azide, with shorter incubation times yielding higher-resolution data. Because the reaction of **Az-1** with ferrous Mb* is fast, short incubation times (<1 min) sufficed for Mb*Int formation and determination of a 1.23 Å resolution structure. Notably, strong electron density in the distal heme pocket allowed modeling of a substrate-derived ligand coordinated to the cofactor. These data confirmed that the azide had undergone a reaction with the heme, eliminating dinitrogen to leave a glycyl moiety bound to the heme iron via its remaining nitrogen (Figure 4a). The distance between the iron and nitrogen of the ligand is 1.97 Å (Table 1), which is significantly shorter than the distance between the iron and N_2_ of the proximal histidine (2.10 Å), indicating a stronger bonding interaction of the iron with the distal ligand. The Fe–N1–C1 angle in Mb*Int is 122.0°, which is close to an ideal trigonal-planar sp^2^ geometry.

**Table 1:**
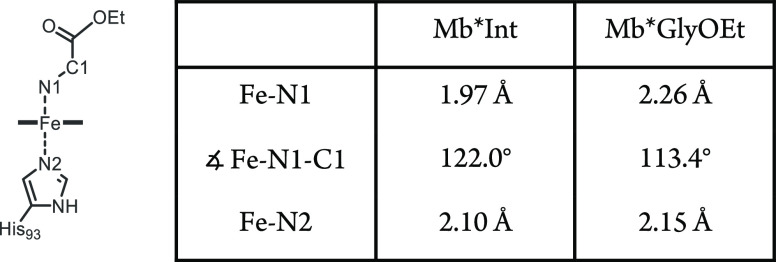
Selected Bond Distances and Bond Angles in Structures of Mb* Treated with **Az-1** or Glycine Ethyl Ester

Analogous experiments were carried out with GlyOEt rather than **Az-1** as a control. Following anaerobic reduction of Mb* crystals and addition of GlyOEt, crystals were captured with a loop, cryoprotected, cryo-cooled, and diffraction data were collected. The best structure had a resolution of 1.39 Å. Again, clear electron density was observed for a ligand in the distal binding pocket, allowing modeling of GlyOEt (Figure S3). In the Mb*GlyOEt complex, the Fe–N1 distance (2.26 Å) is a little longer than the Fe–N2 bond to the proximal histidine (2.15 Å) and, more importantly, substantially longer than the Fe–N1 distance in Mb*Int (Table 1). Interestingly, the Fe–N1 bond distance is also significantly longer than expected for primary amine complexes with organometallic iron (around 2.06 Å),^24^ which presumably reflects constraints imposed by the protein environment. Additionally, the Fe–N1–C1 angle is 113.4°, which is close to the value expected for an sp^3^ geometry. Although the protein backbones of Mb*Int and Mb*GlyOEt closely align with a root-mean-square deviation (rmsd) of 0.115 Å (Figure S4), the differences observed at the active site (Figure 4b) unambiguously demonstrate that the two structures are distinct. In this context, it is worth noting that the same ligand restraints were used for refinement of Mb*Int and Mb*GlyOEt, so the observed differences in bonding geometries can be confidently ascribed to structural differences rather than biases introduced by the refinement process.

### Mössbauer Spectroscopy of the Reactive Intermediate

Mb*Int and Mb*GlyOEt were further characterized by Mössbauer spectroscopy, which offers several advantages over other spectroscopic techniques. Unlike electron paramagnetic resonance (EPR) spectroscopy, it is not limited to paramagnetic compounds and thus reports on all iron species present in a sample, allowing the assignment of both oxidation and spin states. Mössbauer spectroscopy is thus ideally suited for probing the electronic properties of Mb*Int. As Mössbauer spectroscopy on iron requires ^57^Fe, we prepared ^57^Fe heme, extracted the native heme cofactor from Mb*, and reconstituted the apo protein with the ^57^Fe heme to produce ^57^Fe Mb*. The structural integrity of the resulting complex was confirmed by circular dichroism (CD) spectroscopy (Figure S5).

In accord with previous studies on myoglobin, ferric Mb* did not exhibit clearly identifiable Mössbauer signals (Figure S6).^25,26^ Upon reduction of the cofactor with dithionite, however, a spectrum was obtained that consisted of two doublets of similar area (Figure S6). The slightly more abundant species (53%) was fitted to a low-spin Fe(II) heme with an isomer shift of δ = 0.48 mm s^–1^ and a quadrupole splitting of Δeq = 0.95 mm s^–1^ (Table 2). This species was assigned as a hexacoordinate heme with water as the sixth ligand (Mb*(H_2_O)) based on the literature (δ = 0.52 mm s^–1^, Δeq = 1.51 mm s^–1^).^25^ The second species (47% abundance) corresponds to a high-spin Fe(II) species with an isomer shift of δ = 0.91 mm s^–1^ and a quadrupole splitting of Δeq = 2.22 mm s^–1^ (Table 2). These data compare well with literature values for deoxy Fe(II) myoglobin (δ = 0.89 mm s^–1^, Δeq = 2.19 mm s^–1^).^26^

**Table 2:**
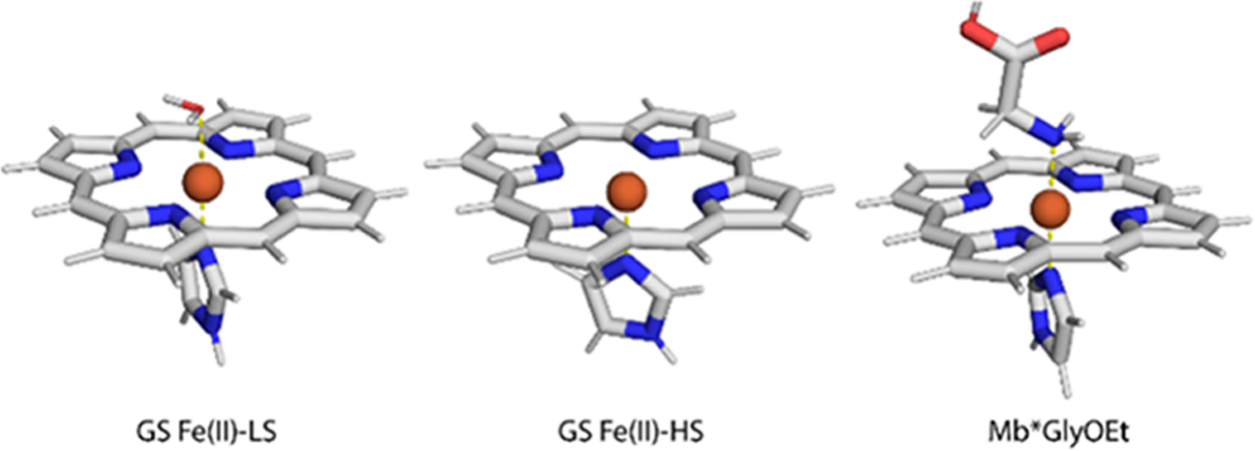
Measured and Calculated Mössbauer Parameters^a^

species	δ (mm s^–1^)	Calc. δ (mm s^–1^)	Δeq (mm s^–1^)	Calc. Δeq (mm s^–1^)
GS Fe(II)-LS	0.48	0.56 (12)	0.95	1.09 (70)
GS Fe(II)-HS	0.91	0.89 (13)	2.22	2.16 (71)
Mb*GlyOEt	0.50	0.52 (12)	1.14	0.63 (70)
Mb*Int	0.38		–0.89	

a The three iron porphyrin complexes shown were structure-optimized using different computational protocols as described in the SI. Then, the isomer shift δ and quadrupole splitting Δeq were calculated for the species using different protocols as described in the SI. This calibration allowed identification of ideal protocols for calculation of the isomer shift δ and quadrupole splitting Δeq. For calculated values, the errors associated with the calculation are given in brackets.

**Figure 5:**
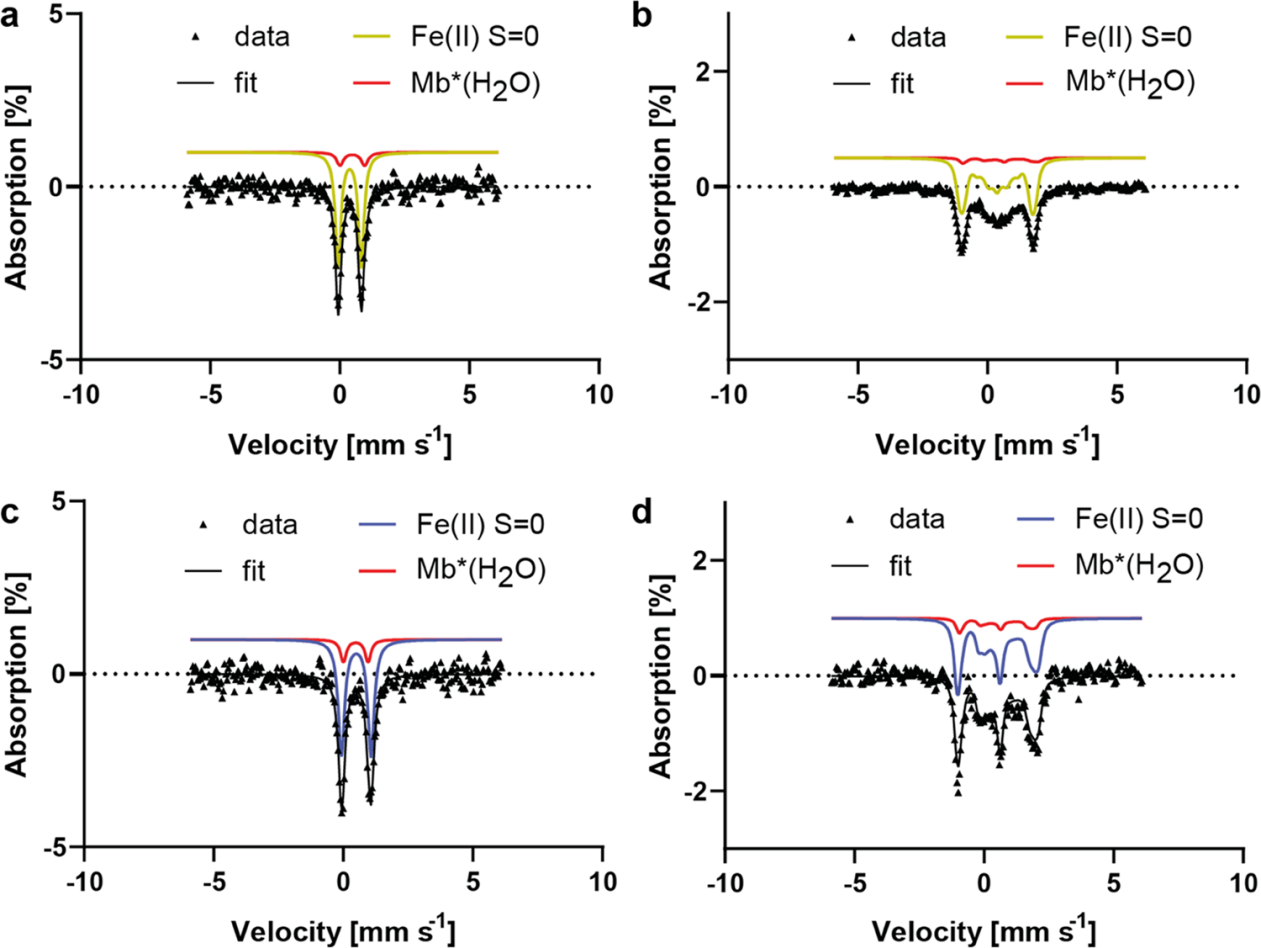
Mössbauer data were collected on Mb*. (a, b) Mössbauer spectra of Mb* + **Az-1** and (c, d) Mössbauer spectra of Mb* + GlyOEt. Mössbauer spectra were recorded at 5.7 K under (a, c) a low (0.06 T) or (b, d) high (7 T) external magnetic field. The presence of shoulders on the peaks and the asymmetry of the lines when a low field was applied indicate the presence of a small quantity of a second species with the same spin state and similar parameters (Figure S7), which we attribute to Mb*(H_2_O) (Figure S6). The spectra of Mb* + **Az-1** were therefore fitted using two different Fe(II) S = 0 species, namely, Mb*Int (yellow) and a minor contribution of Mb*(H_2_O) (red). The spectra of Mb* + GlyOEt were similarly fitted by two Fe(II) S = 0 species assigned to the Mössbauer signatures of Mb* + GlyOEt (blue) and a minor contribution of Mb*(H_2_O) (red).

Addition of **Az-1** to reduced Mb* under argon led to formation of Mb*Int in nearly quantitative yields. This species was identified as an S = 0 Fe(II) species with an isomer shift of δ = 0.38 mm s^–1^ and a quadrupole splitting of |Δeq|= 0.89 mm s^–1^ (Figures 5a, S6, and S7). The fitting process yielded a negative value, but a positive value cannot be excluded. Because of the high asymmetry parameter η, which is close to 1, the sign of the quadrupole splitting is difficult to determine unambiguously. The only other detected species (8%) was the low-spin ferrous aqua complex Mb*(H_2_O).

For comparison, Mössbauer spectra of ferrous Mb*GlyOEt (Figure 5c) appeared as a mixture of two species: a major Fe(II) S = 0 species (85%, spin state determined by high-field Mössbauer spectroscopy), with only a minor contribution (15%) from the hexacoordinate Mb* aqua complex (shown in red as Fe(II) S = 0, II in Figure. 5). The higher fraction of ferrous aqua complex Mb* in this sample compared with the sample incubated with **Az-1** is in line with our UV/vis data that suggest a weaker interaction of GlyOEt with the heme. Like Mb*Int, Mb*GlyOEt was found to be an S = 0 Fe(II) species. However, the isomer shift (δ = 0.50 mm s^–1^) and the quadrupole splitting (Δeq = 1.14 mm s^–1^) are substantially higher than for Mb*Int. At the same time, the asymmetry parameter η (0) is much lower. These data show that Mb*Int and Mb*GlyOEt can be readily distinguished by zero-field Mössbauer spectroscopy.

To confirm the assignment of Mb*Int and Mb*GlyOEt as S = 0 states and to further investigate the differences between the two species, field-dependent Mössbauer experiments were carried out at 7 T (Figure 5b,d). The resulting Mb*Int spectrum is more symmetrical (Figure 5b) than the corresponding spectrum for Mb*GlyOEt (Figure 5d). Clear differences between the spectra include the peak at around 2 mm s^–1^, which is significantly broadened in Mb*GlyOEt, and a sharp feature in Mb*GlyOEt at around 0.5 mm s^–1^ that is absent in Mb*Int.

### Quantum Mechanical Calculations

**Figure 6:**
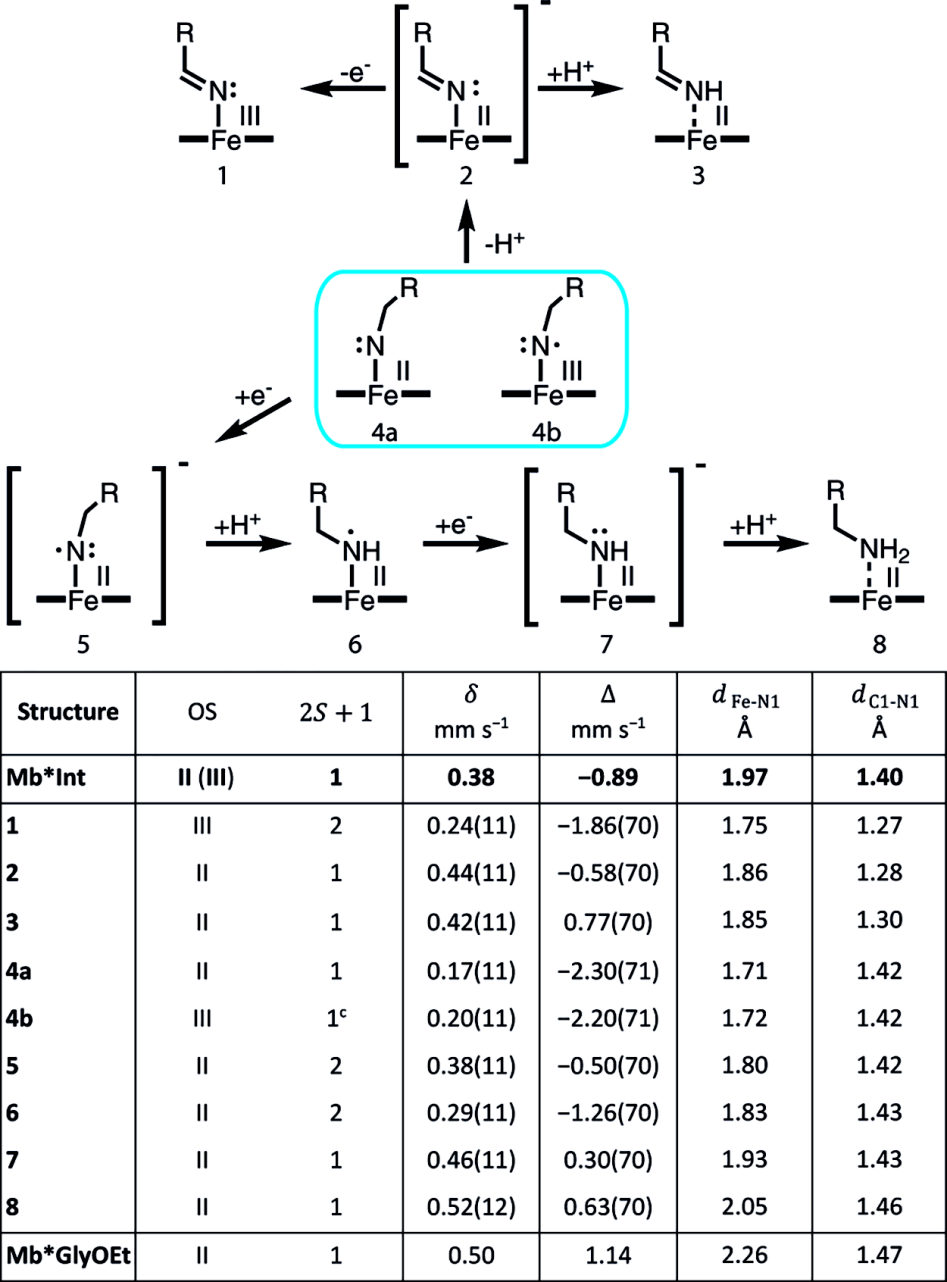
Species considered in the quantum mechanical analysis and calculated and measured values for Mössbauer parameters as well as selected bond lengths. OS, oxidation state; isomer shift, δ; quadrupole splitting, Δ; dFe–N1, distance between the iron center and the adjacent nitrogen; dC1–N1, distance between the nitrogen atom and the adjacent carbon.

UV/vis and Mössbauer spectroscopy suggest that Mb*Int is a ferrous species with a total S = 0. Based on this finding, several possible low-spin systems for Mb*Int were investigated, as shown in Figure 6. For the calculations, simplified iron porphyrin systems with an imidazole ligand were used (SI). We considered two possible nitrene species: a ferrous nitrene (**4a**; total spin, S_tot_ = 0 as both S_Fe(II)_ = 0 and S_nitrogen_ = 0) and a ferric species antiferromagnetically coupled to a nitrogen radical (**4b**; *S*_tot_ = 0 resulting from S_Fe(III)_ = 1/2 coupled to S_nitrogen_ = 1/2). Furthermore, we considered various ferrous species that could lie on the reaction coordinate for azide reduction to an amine, such as an anionic ferrous species with a radical on the nitrogen (**5**; *S*_tot_ = 1/2 as S_Fe(II)_ = 0 and S_nitrogen_ = 1/2), a neutral ferrous species with an associated nitrogen radical arising from protonation of species **5** (**6**; *S*_tot_ = 1/2 as S_Fe(II)_ = 0 and S_nitrogen_ = 1/2), another anionic ferrous species resulting from a one-electron reduction of **6** (**7**; *S*_tot_ = 0 as both S_Fe(II)_ = 0 and S_nitrogen_ = 0) as well as the fully reduced ferrous amine corresponding to Mb*GlyOEt (**8**; *S*_tot_ = 0 as both S_Fe(II)_ = 0 and S_nitrogen_ = 0). Additionally, we also looked at several imine species that could arise after an initial deprotonation step of a nitrene intermediate: an anionic ferrous imine (**2**; *S*_tot_ = 0 as both S_Fe(II)_ = 0 and S_nitrogen_ = 0), a ferric imine (**1**; *S*_tot_ = 1/2 as S_Fe(III)_ = 1/2 and S_nitrogen_ = 0), and a ferrous imine (**3**; *S*_tot_ = 0 as both S_Fe(II)_ = 0 and S_nitrogen_ = 0).

Before the Mössbauer parameters were calculated, structure optimizations for all hypothetical structures were carried out and compared to the experimental values from the crystal structure (Figure 6). The Fe–N1 bond length of 1.97 Å measured in the crystal structure of Mb*Int is very long compared to typical organometallic nitrene complexes, which are characterized by metal–nitrogen bonds shorter than 1.80 Å.^24^ For example, a complex reported by the Betley group in which a high-spin Fe(III) is antiferromagnetically coupled to a nitrogen-based radical has an Fe–N bond distance of 1.77 Å.^27^ Consistent with this precedent, the ferrous nitrene **4a** and the ferric heme antiferromagnetically coupled to the nitrogen radical **4b** were calculated to have Fe–N1 bond distances of 1.71 and 1.72 Å, respectively. In contrast, structures of several hypothetical intermediates that could be obtained by reduction of the nitrene were found to have Fe–N1 bond lengths within a standard deviation of the longer measured bond length of 1.97 Å for Mb*Int (Figure 6, Table S7). These bond lengths are in good agreement with those reported for previously characterized metal amide anions.^28^ The measured bond angles for the ligand in the protein crystal structure, which are around 120°, provide additional support for an amide structure, as previously reported nitrenes have nearly linear binding geometries with Fe–N1–C1 binding angles above 150°.^27,29^

To identify a suitable computational protocol for the calculation of Mössbauer parameters, we benchmarked different computational protocols using three structures—the water-bound resting state, the apo resting state, and the iron porphyrin complexed with glycine (which served as a surrogate for glycine ethyl ester (Table 2))—and compared the values to the experimental Mössbauer parameters for the two species obtained for reduced Mb* and Mb*GlyOEt. Two different protocols were considered for structure optimization (protocol “a” used a PBE exchange correlation functional,^11,30^ and protocol “b” used a TPSS exchange correlation functional^31^) and two different protocols for property calculations (protocol “α” used a PBE0 exchange correlation functional,^11,30^ and protocol “β” used a TPSSh exchange correlation functional^31^) (see Table S3). The performance of the resulting four possible combinations (α/a, β/a, α/b, and β/b) was tested on the three reference systems. None of the protocols were able to reproduce the experimental values of both the isomer shift and quadrupole splitting for all reference systems. However, the α/a protocol consistently reproduced the experimental isomer shift, whereas the β/a protocol consistently reproduced the experimental quadrupole splitting (Tables S5 and S6). Consequently, isomer shifts for the candidate structures were obtained with a computational protocol different from that of the corresponding quadrupole splittings (Tables 2 and S7).

Having established optimal protocols for calculating the isomer shifts and quadrupole splittings, we calculated Mössbauer parameters for eight species (**1**–**3**, **4a, 4b**, **5**–**7**) with different electronic properties that we considered possible candidates for the Mb*Int intermediate (Figure 6). We identified three low-spin Fe(II) structures for which the protocols reproduced the experimental Mössbauer parameters and the axial Fe–N1 bond length of interest with 95% confidence, namely, the anionic imine **2**, the neutral imine **3**, and the anionic amide **7** (Figure 6, Table S7). Although calculations for the S = 1/2 species **5** and **6** reproduced the measured Mössbauer parameters and the Fe–N1 bond length for Mb*Int, the intermediate is an S = 0 species as clearly identified by field-dependent Mössbauer experiments. Therefore, we exclude these structures as potential representations of Mb*Int. Structures of imines **2** and **3** can also be excluded because the C1–N1 bond lengths in their computationally optimized structures are 1.28 and 1.30 Å, respectively, which are significantly shorter than the measured bond length in Mb*Int (1.40 Å). Compiling all of the data, we therefore conclude that the most likely representation of the observed structure Mb*Int is the anionic amide **7**, which has calculated Mössbauer parameters and Fe–N1 and C1–N1 bond lengths in good agreement with the experiment.

## Discussion

**Figure 7:**
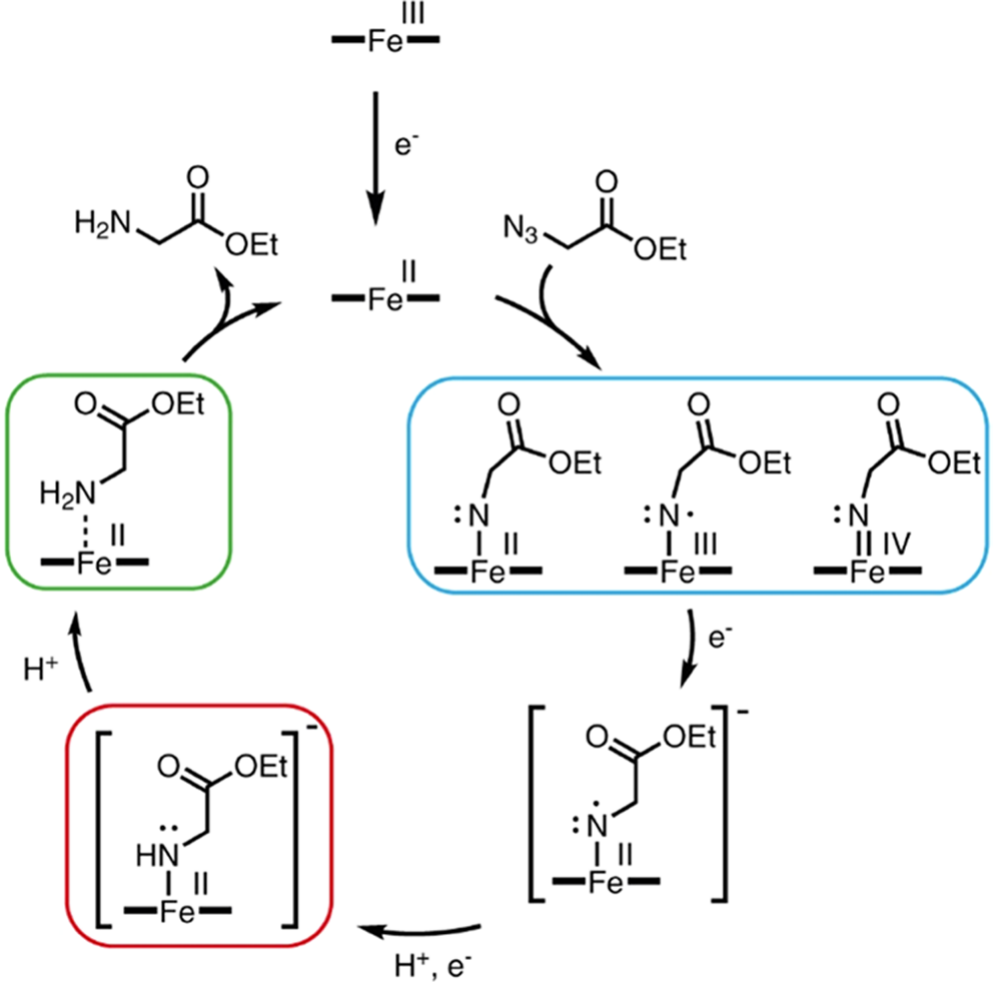
Proposed catalytic cycle for azide reduction by myoglobin. Possible nitrene structures are shown in the blue box, the isolated intermediate Mb*Int (**7**) in the red box, and Mb*GlyOEt (**8**) in the green box. The order of the protonation and electron transfer steps leading to Mb*Int has not been established unambiguously.

Mechanistic studies on nitrene transfer reactions catalyzed by P450s, ruthenium porphyrins, and iron complexes suggest that C–H amination proceeds via a radical rebound pathway with hydrogen atom abstraction as the rate-determining step.^1,3,27,28,32,33^ Myoglobin-catalyzed cyclization of arylsulfonyl azides exhibits a similar kinetic isotope effect as cytochrome P450 and likely follows a similar reaction mechanism.^1,17^ Based on this hypothesis, our NMR, UV/vis, Mössbauer, and crystallographic data, as well as previous work from others,^1,3,4,20,21^ we therefore propose the pathway shown in Figure 7 for the reaction of Mb* with azides that cannot undergo intramolecular cyclization.

As ferric Mb* is unreactive toward azides, the catalytic cycle must be initiated by the reduction of the heme cofactor. Ferrous Mb* then reacts with an azide like **Az-1** to form a transient nitrene intermediate, which can be formulated as an Fe(IV) imido, an Fe(III) imidyl radical, or an Fe(II) nitrene adduct. Unlike structurally analogous carbene intermediates,^11,34^ this species does not accumulate, precluding direct characterization. If the hypothesized radical rebound pathway for myoglobin is correct, the enzyme-bound nitrene intermediate is probably best represented as a nitrogen radical that is antiferromagnetically coupled to the Fe(III) heme. Radical species are not very stable in myoglobin, however. For example, in contrast to P450s and other heme proteins, compound I, a highly reactive ferryl oxo cation radical, does not accumulate in myoglobin.^14^ Due to the relatively high reduction potential of Mb* (+30 mV)^11^ and the presence of excess dithionite in the reaction mixture, the nitrogen radical would be expected to undergo rapid reduction unless trapped by the substrate itself in a competitive intramolecular cyclization reaction. Two one-electron reductions and a protonation step would then yield Mb*Int, the S = 0 ferrous amide anion that we captured and characterized in our experiments (Figure 7, boxed in red). Since Mb*Int is the only intermediate observed in the reduction reaction under turnover conditions, we suggest that protonation of this species to give Mb*GlyOEt is the rate-determining step. Dissociation of the product from the active site then regenerates ferrous Mb*, which can initiate a new reaction cycle.

Previous investigations of heme-derived catalysts with reduction potentials between −900 and −300 mV had suggested that the best catalysts for nitrene transfer had reduction potentials at the upper end of this range.^29,35^ For instance, a P450 variant with an axial serine ligand instead of the native cysteine has an Fe(III)/Fe(II) reduction potential of −293 mV and was converted into an efficient nitrene transferase by introducing only three active site mutations.^4,36^ Although myoglobin has a reduction potential that is substantially higher than that of P450 or other cytochromes, it is a poor nitrene transferase for intermolecular reactions. These findings suggest that there may be a “sweet spot” where the reduction potential is sufficiently high to allow efficient generation of the iron nitrene but low enough to prevent its fast reduction by excess reductant. Tuning the reduction potential of myoglobin by incorporating noncanonical axial ligands or using synthetic porphyrin cofactors^11,12,37−39^ might therefore yield variants that are more effective catalysts for intermolecular nitrene transfers. In the case of intramolecular reactions,^17,40^ such tuning is presumably less important because preorganization of the substrate in the active site allows cyclization to effectively compete with over-reduction.

Avoiding agents such as dithionite that can reduce the nitrene intermediate might be an interesting alternative strategy. Although such an approach might suffer from the slow formation of active nitrene species, myoglobin variants with even higher reduction potentials might be useful for the generation of stable nitrene complexes. We recently reported a myoglobin variant with a noncanonical proximal thiazole ligand, which forms an unusually stable ferric oxymyoglobin complex in the absence of a reductant.^12^ As such complexes are best characterized as ferriheme-superoxide species that are similar to the Fe(III) imidyl radicals required for nitrene transfer, this protein could be an attractive candidate for nitrene transfer.

## Conclusions

In this work, we have captured and characterized a reactive anionic ferrous intermediate in the reduction of azides to amines catalyzed by heme proteins. The insights gained from this study may inspire strategies for preventing this often-undesirable side reaction and foster the development of myoglobin variants capable of catalyzing otherwise challenging intermolecular nitrene transfer reactions.

## Supporting Information Available

Materials and methods; kinetic measurements of intermediate formation upon fast mixing of Mb* and **Az-1**; UV/vis spectrum of Mb*-O_2_; CD spectrum comparing Mb* and reconstituted ^57^Fe-Mb*; data collection and refinement statistics of myoglobin nitrenoid and amine complexs; sample preparation for Mössbauer spectroscopy; results of broken-symmetry calculations; and computational details (PDF)
