# Supplementary material for: Myoglobin-Catalyzed Azide Reduction Proceeds via an Anionic Metal Amide Intermediate
Source: J Am Chem Soc. 2024 Jan 9;146(3):1957–66. doi: 10.1021/jacs.3c09279 (PMC10811658; doi:10.1021/jacs.3c09279)
Supplement: Supplementary file 1 — ja3c09279_si_001.pdf [file ja3c09279_si_001.pdf]

Supporting Information for

Myoglobin-catalyzed azide reduction proceeds via an anionic metal amide intermediate

AUTHORS

Matthias Tinzl[1], Johannes V. Diedrich[2], Peer R. E. Mittl[3], Martin Clémancey[4], Markus Reiher[5], Jonny Proppe[2], Jean-Marc Latour[4], Donald Hilvert[1,*]

[1]Laboratory of Organic Chemistry, ETH Zürich, 8093 Zürich, Switzerland

[2]Institute of Physical and Theoretical Chemistry, TU Braunschweig, 38106 Braunschweig, Germany

[3]Department of Biochemistry, University of Zürich, 8057 Zürich, Switzerland

[4]Univ. Grenoble Alpes, CNRS, CEA, IRIG, Laboratoire de Chimie et Biologie des Métaux, 17 Rue des Martyrs, Grenoble, Cedex F-38054, France

[5]Institute for Molecular Physical Science, ETH Zürich, 8093 Zürich, Switzerland

*hilvert@org.chem.ethz.ch

Experimental Methods	S2-S6
Supplementary Figures S1-S9	S7-S14
Supplementary Tables S1-S8	S15-S22
Synthetic Procedures	S23-S25
Computational Details	S26-S30
Appendix	S31
Bibliography	S32-S34

(A.1) DNA and Protein Sequences. Mb H64V V68A (Mb*) was previously cloned into a pET29b vector as described in ref [1].

The DNA sequence of Mb*:

5'-ATGATGGTTCTGTCTGAAGGTGAATGGCAGCTGGTTCTGCATGTTTGGGCTAAAGTTGAAGCTGAC
GTCGCTGGTCATGGTCAGGACATCTTGATTCGACTGTTCAAATCTCATCCGGAAACTCTGGAAAAATT
CGATCGTTTCAAACATCTGAAAACTGAAGCTGAAATGAAAGCTTCTGAAGATCTGAAAAAAGTGGGTG
TTACCGCGTTAACTGCCCTAGGTGCTATCCTTAAGAAAAAAGGGCATCATGAAGCTGAGCTCAAACCG
CTTGCACAATCGCATGCTACTAAACATAAGATCCCGATCAAATACCTGGAATTCATCTCTGAAGCGAT
CATCCATGTTCTGCATTCTAGACATCCAGGTGACTTCGGTGCTGACGCTCAGGGTGCTATGAACAAAG
CTCTGGAGCTGTTCCGTAAAGATATCGCTGCTAAGTACAAAGAACTGGGTTACCAGGGTGGCTCGGGA
CATCATCACCATCACCATTGA

The amino acid sequence of Mb*:

MMVLSEGEWQLVLHVWAKVEADVAGHGQDILIRLFKSHPETLEKFDRFKHLKTEAEMKASEDLKKVGV
TALTALGAILKKKGHHEAELKPLAQSHATKHKIPIKYLEFISEAIIHVLHSRHPGDFGADAQGAMNKA
LELFRKDIAAKYKELGYQGGSGHHHHHH

(A.2) Myoglobin protein production and purification.[2] For expression of Mb*, pET29b_Mb H64V V68A was transformed into BL21(DE3) *E. col* and the cells were plated on a LB agar plate containing 50 µg/ml kanamycin. A single colony of freshly transformed cells was cultured overnight in 3 ml of LB medium containing 50 µg/ml kanamycin. 1 ml of the culture was used to inoculate 100 mL of 2xYT medium supplemented with 1 mM δ-aminolevulinic acid (1M stock) and 50 µg/ml kanamycin. The culture was incubated for ~2 h at 37 °C at a shaking speed of 230 rpm. When the OD_{600} of the culture reached 0.4 to 0.5, IPTG was added to a final concentration of 0.1 mM to induce expression of the Mb* protein. The induced cultures were incubated for ~24 h at 25 °C, and the cells were subsequently harvested by centrifugation at 8,000 *g* for 10 min. The pelleted bacterial cells were resuspended in PBS buffer (Na_2HPO_4 10 mM, KH_2PO_4 1.8 mM, NaCl 137 mM, KCl 2.7 mM pH 7.4) and sonicated to disrupt the cells. To maximize heme occupancy, Mb* and Mb*NMH were reconstituted with hemin chloride. Briefly, the lysate solutions were mixed with 0.2 mM of hemin chloride (20 mM stock solution in 10 mM NaOH), and incubated at 4 °C for 10 min. The lysate was centrifuged at 11,500 *g* for 15 min and the supernatant was subjected to affinity chromatography using Ni-NTA Agarose (Qiagen, Helden, Germany). After two washes with buffer containing 50 mM Tris-HCl, 300 mM NaCl and 10 mM imidazole at pH 8.0, the proteins were eluted by 50 mM Tris-HCl, 300 mM NaCl at pH 8.0 containing 300 mM imidazole. The protein solutions were then subjected to size-exclusion (SEC) chromatography on Superdex 75 10/300 increase GL (GE healthcare Life Sciences) with a flow rate of 0.7 ml/min using an NGC Quest 10 plus FPLC system equilibrated with PBS. The fractions were monitored at 260, 280, and 408 nm, collected using a BioFrac fraction collector, and analyzed by SDS-PAGE. If necessary, the

protein solution was concentrated by Amicon Ultra-15 10 kDa cut-off centrifugal filter device (Merck Millipore, Massachusetts, USA). The concentrations of heme proteins were determined by UV-vis absorbance measurements at 407 nm using an extinction coefficient ε_{407} = 146,000 $M^{-1}cm^{-1}$ as previously reported determined.[1]

(B) Crystallization and structure determination of myoglobin. Mb* in PBS buffer was concentrated to approximately 45 mg/ml using an Amicon Ultra-15 10 kDa cut-off centrifugal filter device. The protein solution was passed through a Zeba spin desalting column pre-equilibrated with pure water, and the final concentration was adjusted to 30 mg/ml. The protein was crystallized by vapor diffusion in sitting drops at 20 °C. An initial crystallization hit was identified in a well containing 0.1 M succinic acid, sodium dihydrogen phosphate glycine (SPG) (molar ratio 2:7:7) buffer at pH 7.0 containing 25% (w/v) PEG1500 by using the commercial The PACT Suite sparse matrix screen (Qiagen). Crystallization conditions were improved by varying the pH and the concentration of PEG 1500. The optimal crystallization conditions were found to be 0.1 M SPG buffer at pH 6.8-7.2 containing 28-32% (w/v) PEG 1500 and mixing equal volumes (0.2 µl) of protein and well solution.

<u>Ferrous amine and ferrous amide in Mb*:</u>

Crystals were grown as described above. Crystals from approximately 15 wells were pooled in ca. 200 µL SPG buffer (pH 7.0) containing 30% (w/v) PEG1500 in a Schlenk tube. The solution was made anaerobic by performing at least 5 cycles of degassing and nitrogen gas purging. To produce the intermediate, a few drops of an anaerobic stock of dithionite (1 M) in buffer and a few drops of azide/amine solution (400 mM) in methanol/buffer were added with a syringe. Crystals were soaked for ca. 30 seconds, then removed with a syringe, placed in a petri dish, and captured with a suitable loop. Typically, crystals were incubated with azide/amine for variable lengths of time, ranging from less than 1 min up to 15 min; longer incubation times led to disintegration of the crystals. The crystals were subsequently cryoprotected by dipping them in degassed SPG buffer (pH 7.0) containing 20% (w/v) PEG1500 and 20% (w/v) ethylene glycol. Afterward, the crystals were flash-cooled in liquid nitrogen. Importantly, the steps after removing the crystals from the Schlenk tube were carried out as rapidly as possible (typically within 5-10 seconds). Diffraction data were collected at 100 K using a wavelength of 1.0000 Å at the Swiss Light Source (SLS). The structures were solved by molecular replacement using a myoglobin structure (PDB ID: 1A6K) and further refined using the program PHENIX[3] and coot[4]. Data collection and refinement statistics are shown in Table S1. For refinement, the protein backbone was first refined without placing waters, the heme cofactor or ligands in the model. These were introduced in subsequent rounds of refinement. The topology file for the ligand

was created using the prodrg server. For both structures the same topology file was used. Raw data is deposited on protein https://www.proteindiffraction.org.

(C) Biocatalytic reduction of azides by myoglobin and NMR. Reactions were typically run on a 400 µL scale in 2 mL sealed glass vials. The enzyme (final concentration 10 µM) was added to degassed 50 mM potassium phosphate buffer pH 8.0 and the solution was made anaerobic by continuous headspace exchange with wet nitrogen gas for a minimum of 10 minutes. The reactions were initiated by adding various amounts of dithionite (400 mM stock solution in 50 mM potassium phosphate buffer pH 8.0), and organic azides (10 µL of 800 mM stock). The reactions were stirred for 1 h at 230 rpm using a magnetic stirring bar and either quenched with 100 µL of 3 M hydrochloric acid and centrifuged at 15,000 g for 5 minutes to remove the enzyme or alternatively filtered through a syringe filter to prevent hydrolysis of esters. The supernatant or filtered solution was analyzed by LC-MS. For Az-1, the only compound detected corresponded to the amine, whereas for Az-2, both the corresponding amine and 2,5-diethyl pyrazine were detected. Compounds were identified by comparison to authentic standards.

For NMR experiments, 800 µL of a 50 µM enzyme solution in 50 mM potassium phosphate buffer in D_2O was made anaerobic by continuous headspace exchange with wet nitrogen gas for a minimum of 10 minutes. The solution was then anaerobically transferred into an NMR tube sealed with an air-tight rubber stopper. The reactions were initiated by adding 20 µL dithionite (400 mM stock in 50 mM potassium phosphate buffer in D_2O), and organic azide (10 µL of 800 mM stock in D_6-DMSO).

(D) UV/Vis measurements myoglobin. All spectra were recorded anaerobically in 50 mM potassium phosphate buffer at pH 8.0 unless indicated otherwise. Typically, a solution of 200-800 µl containing 0.1-10 µM concentrations of Mb* was transferred to a cuvette (either 2 mm or 1 cm width depending on Mb* concentration) which was sealed with a teflon stopper and the headspace was vigorously exchanged under nitrogen flow for at least 10 minutes. An initial spectrum was recorded to confirm that Mb* was in its ferric state. Subsequently, dithionite was added anerobically to the solution (from an anerobic 400 mM stock solution), final concentrations were typically between 100 µM- 2.5 mM. In order to confirm full formation of ferrous Mb*, a spectrum was recorded immediately after dithionite addition. Then, either azide or GlyOEt was added to the solution and spectra were recorded within less than 1 min after addition. Overall, all additions and measurments were typically completed within 10 minutes.

For measuring Mb*-O_2, Mb* sample was reduced by addition of excess dithionite. Then, excess dithionite was removed by running the sample over a short PD-10 desalting column. The sample was quickly diluted in buffer saturated with oxygen by previous bubbling with nitrogen gas.

(E) Stopped-flow kinetics. The rate of formation of Mb*Int was measured using ultrafast kinetics in a stopped-flow device. To that end, an anerobically reduced solution of myoglobin (20 µM, 20 mM dithionite) was mixed in a 1:1 ratio with solutions of 10-100 mM Az-1 in 50 mM potassium phosphate pH=8.0 and 10 % methanol. The increase in absorbance was monitored at 425 nm and fitted to an exponential function.

(F) Pyrrole formation. Reactions were run on a 400 µL scale in 2 mL sealed glass vials. The enzyme (final concentration 20 µM) was added to degassed 50 mM potassium phosphate buffer pH 8.0 and the solution was made anaerobic by continuous headspace exchange with wet nitrogen gas for a minimum of 10 minutes. The reactions were initiated by adding variable amounts of dithionite (400 mM stock solution in 50 mM potassium phosphate buffer pH 8.0), azide (10 µL of 800 mM stock in methanol) and acetoacetate (20 µL of 4 M stock). The reactions were stirred overnight at 230 rpm using a magnetic stirring bar. Precipitation of the protein was observed in the course of the reaction. For product quantification, 100 µL of 10 mM pyrrole was added as standard and the product was extracted with 900 µL ethyl acetate. The product was quantified by GC-FID analysis using calibration curves prepared from authentic standards.

(G) Mössbauer. Myoglobin was enriched in ^{57}Fe heme by extraction of the cofactor and reconstitution with ^{57}Fe heme. A typical extraction was carried out by adding 50 µL of 1 M hydrochloric acid to 0.5 mL protein solution (conc. 50-100 µM) in a falcon tube. The pH was checked to ensure that it was lower than 4. Then 1.5 mL PBS was added and the porphyrin was extracted with pentanone (2 x 2 mL). To allow faster phase separation, the falcon tube was centrifuged for 30 s at low speed. The organic phase was discarded and the resulting white aqueous phase was checked for the presence of heme by Nanodrop. When no heme was detected, approximately 1 mL of a ^{57}Fe heme solution (5-10 mg ^{57}Fe heme dissolved in 1 mL 0.1 M NaOH that was then diluted with 9 mL water) was added to the protein solution. After mixing, 5 mL of 50 mM potassium phosphate buffer (pH=7.4) was added. Purification by Ni-NTA (as described earlier) and buffer exchange to 20 mM MOPS buffer (pH=7.0) using a PD-10 columns afforded ^{57}Fe-Mb* in approximately 50% yield. The integrity of the protein was checked by UV/Vis and CD spectroscopy (Figure S5). Samples for Mössbauer spectroscopy were prepared under argon according to Table S2 using proteins in 20 mM MOPS buffer (pH=7.0). Typically, between the addition of azide and freezing the sample for characterization in liquid nitrogen, less than 5 minutes elapsed which corresponds to the timescales of the UV/vis spectroscopy experiments. Mössbauer spectra were recorded at 5.7 K and under magnetic fields of 0.06 or 7 T applied parallel to the γ beam on a strong-field Mössbauer spectrometer equipped with an Oxford Instruments Spectromag 4000 cryostat containing an 8 T split-pair superconducting magnet. High field experiments were used to

determine the spin state of the species. The spectrometers were operated in a constant acceleration mode in transmission geometry. The isomer shifts were referenced against that of a metallic iron foil at room-temperature. Analysis of the data was performed with the software WMOSS Mössbauer Spectral Analysis Software (www. wmoss.org, 2012–2013, Web Research, Edina). For the spectra of Mb*+Az-1 and Mb*+GlyOEt (Fig 5), fits assuming only a single species were unsatisfactory (Fig. S5b) and improved significantly upon introduction of Mb*(H_2O) as a minor species.

Supplementary Figures:

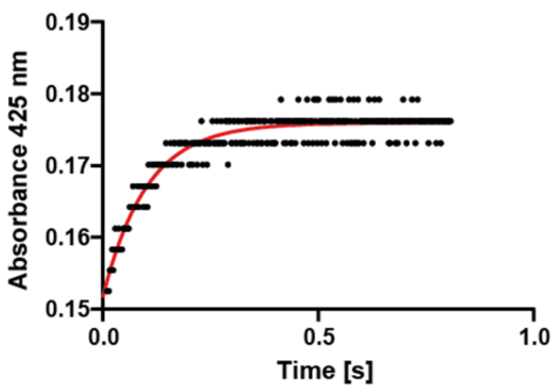

Figure S1: Kinetic measurements of intermediate formation upon fast mixing of Mb* and **Az-1**. Mb* which was anaerobically reduced by dithionite was mixed with **Az-1** and formation of the new species was monitored at 425 nm. The half-life was calculated to be $t_{1/2}$ ~0.07 s at 50 mM Az-1 and $t_{1/2}$ ~0.25 s at 5 mM Az-1.

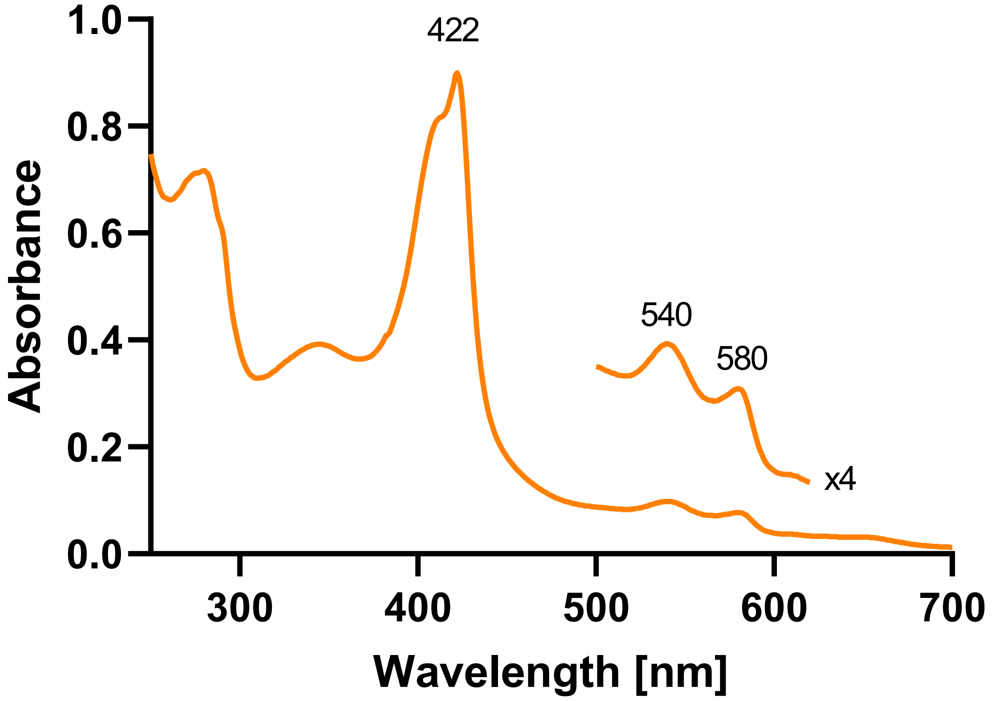

Figure S2: UV/Vis spectrum of Mb*-O_2. Absorption maxima are found at 422, 540 and 580 nm. The shoulder at around 410 nm indicates that met-Mb* has already started to form illustrating the low stability of Mb*-O_2 and suggesting fast autooxidation.

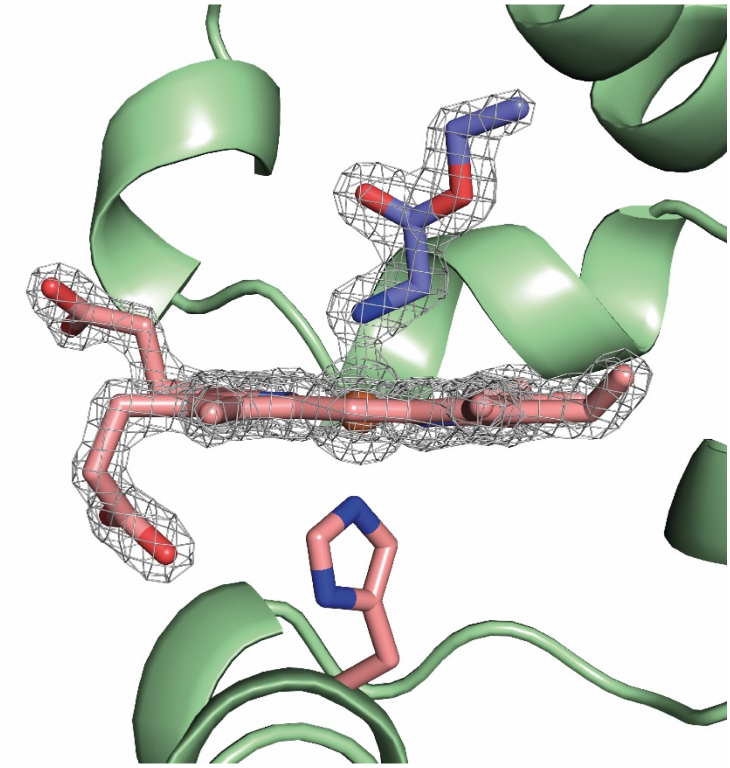

Figure S3 Structure of Mb* soaked with glycine ethyl ester (purple). The F_o-F_c omit map (gray mesh) was contoured at 3σ.

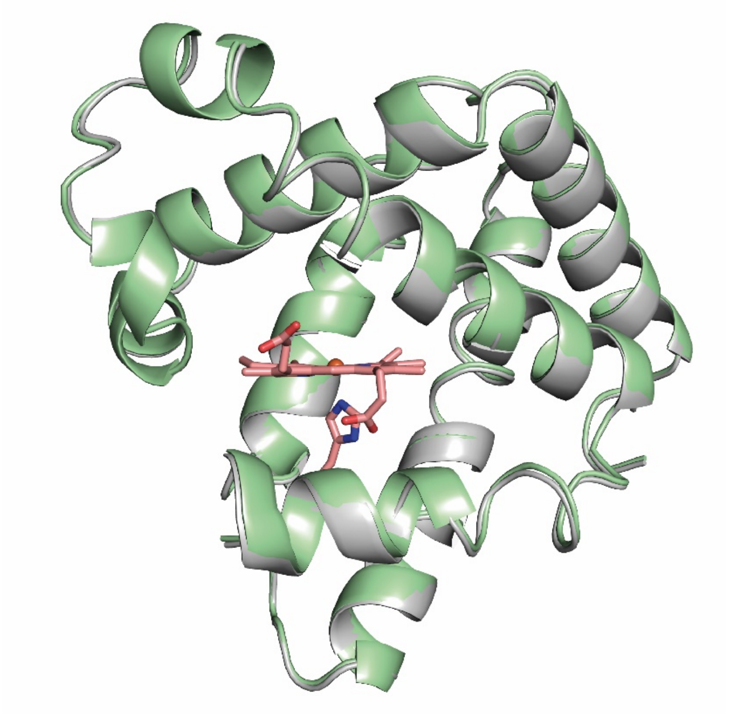

Figure S4 Overlay of structures obtained by soaking Mb* with azide (gray cartoon) or glycine ethyl ester (green cartoon).

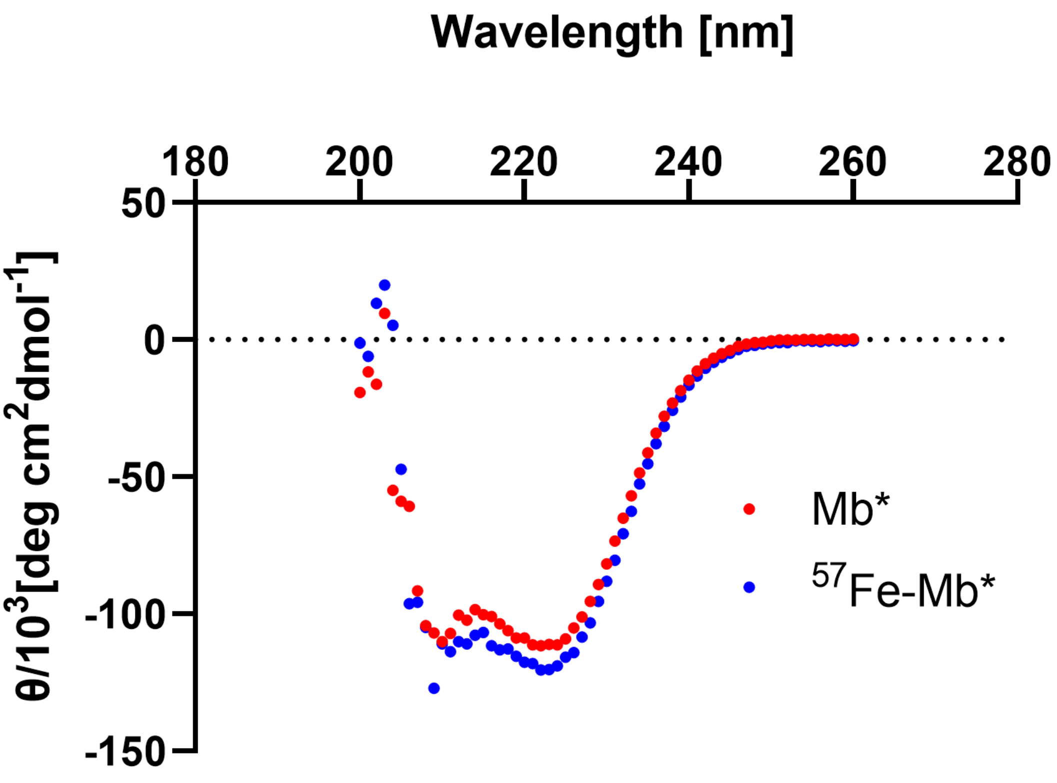

Figure S5: CD spectrum comparing Mb* and reconstituted ^{57}Fe-Mb*.

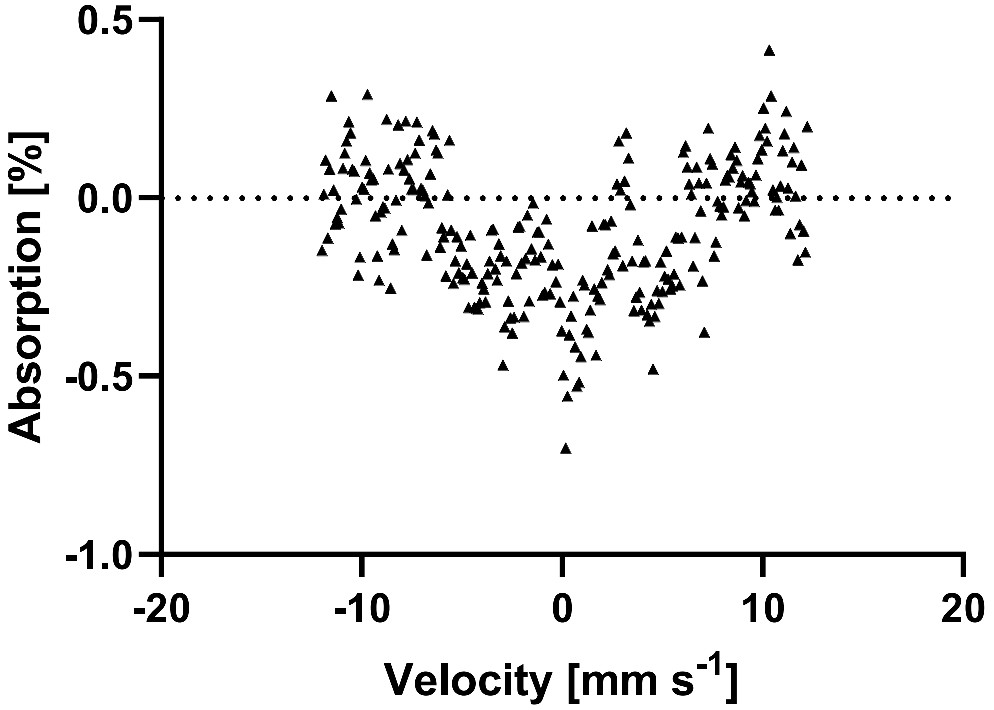

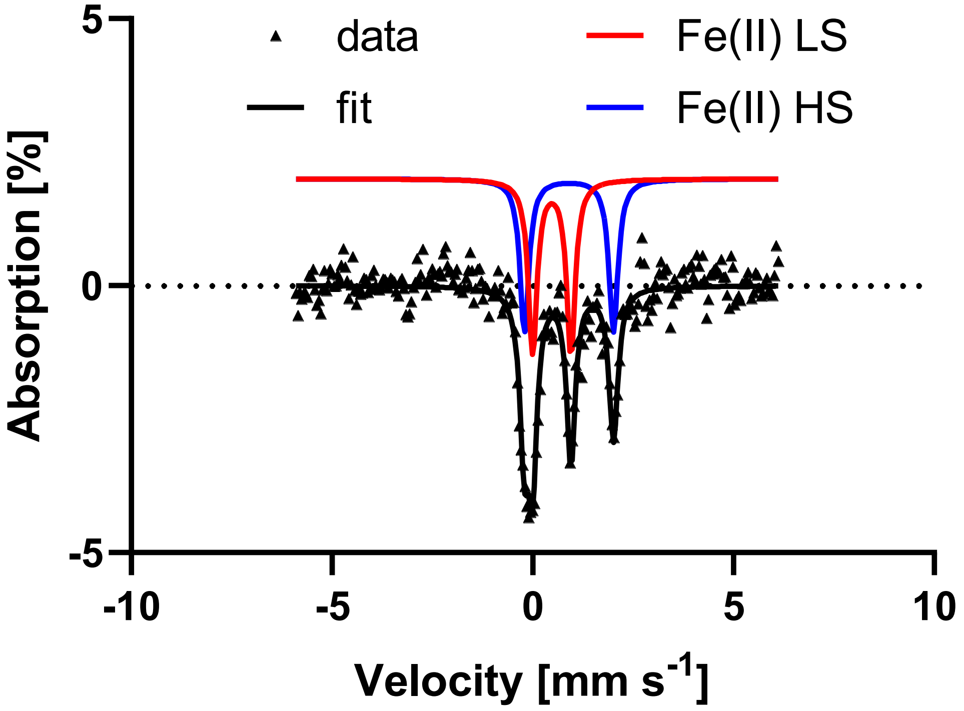

Figure S6: Mössbauer spectra of oxidized Mb* and Mb* reduced with dithionite. While oxidized myoglobin did not produce clearly distinguishable Mössbauer signals, reduced Mb* showed two doublets of similar area. Fe(II) HS = pentacoordinate Mb*(δ = 0.91 mm s^{-1}, ΔEq = 2.22) and Fe(II) LS = hexacoordinate Mb*(H2O) *(δ = 0.48 mm s^{-1}, ΔEq = 0.95), which is in good agreement with previous reports.[5,6]

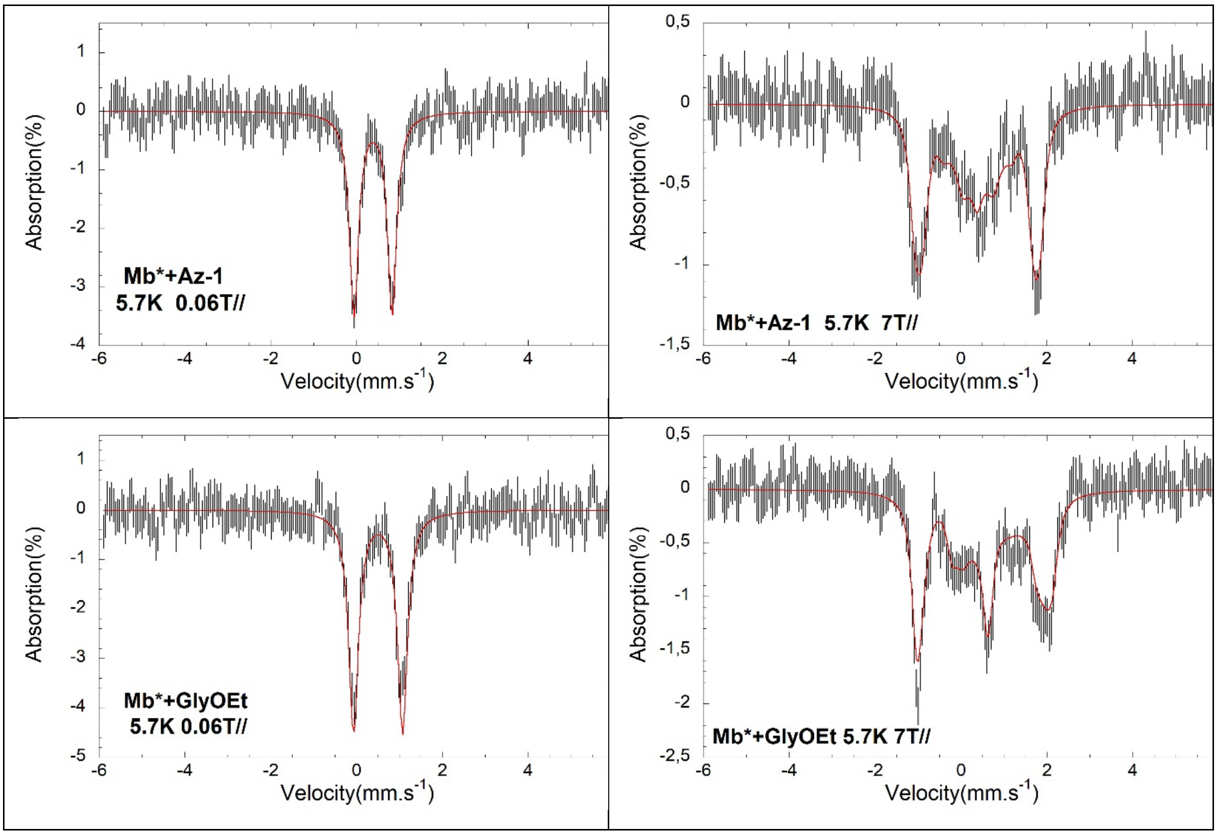

Figure S7: Simulations (red curves) of the Mössbauer spectra (hashed bars) of Mb* + **Az-1** (top) and Mb*+GlyOEt (bottom) recorded at 5.7 K with a small (0.06 T, left) or a high (7 T, right) magnetic field applied parallel to the γ beam, assuming a unique species. The spectrum in the top left panel shows a shoulder at ca. 1 mm·s^{-1} that is not reproduced by the simulation. Similarly, the asymmetry of the two lines in the spectrum in the bottom left panel is not reproduced by the simulation. Both features are reproduced when the presence of a small amount of Mb*(H_2O) is included, as illustrated in Figure 5 in the maintext.

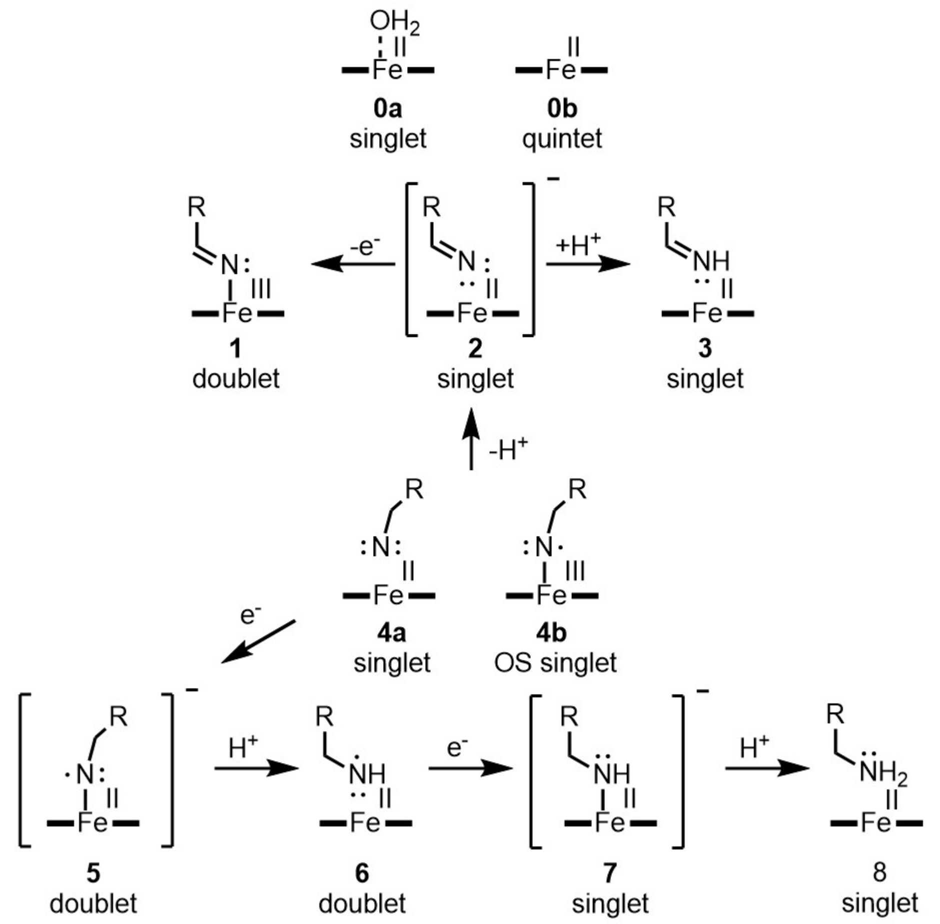

Figure S8: Schematic structures of investigated species ordered according to subsequent reduction and protonation steps. See Figure C2 for more details. Species **4b** is represented as a broken-symmetry (BS) solution.

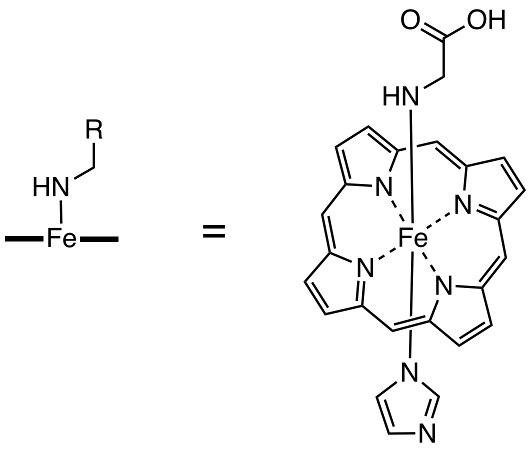

Figure S9: Graphical abstraction of the structural motifs shown in Figure S8.

Supplementary Tables:

Table S1: Data Collection and refinement statistics of myoglobin nitrenoid and amine complexs.

	Mb*+**Az-1**	Mb*+glycine ethyl ester
PDB entry	8QBC	8QBA
Number of crystals	1	1
**Data collection**		
X-ray wavelength (Å)	1.0000	1.0000
Rotation range (°)	0-180	0-180
$\Delta\ \phi$ (°)	0.1	0.1
Space group	$P2_12_12_1$	$P2_12_12_1$
Cell dimensions		
a, *b*, *c* (Å)	39.8, 47.2, 76.2	39.9, 47.8, 77.4
Resolution (Å)	38.1-1.23 (1.27-1.23)	38.7-1.39 (1.44-1.39)
R_{merge}	2.1 (63.7)	2.1 (51.8)
$I / \sigma I$	20.0 (1.04)	17.18 (1.52)
CC (1/2) (%)	100 (47.1)	100 (68.4)
Completeness (%)	96.5 (72.1)	99.9 (99.8)
Redundancy	1.9 (1.6)	2.0 (2.0)
**Refinement**		
No. reflections	40993	30519
R_{work} (%)	14.51	14.62
R_{free} (%)	18.00	19.90
No. atoms	1611	1540
Protein	1314	1283
Ligand/ion	58	54
Water	239	203
B-factors (Å^2)		
Protein	16.2	23.5
Ligands	17.7	21.3
Water	35.0	46.7
R.m.s. deviations		
Bond lengths (Å)	0.013	0.005
Bond angles (°)	1.28	0.80
Ramachandran plot (%)		
Favored	97.4	97.4
Allowed	2.6	2.6
Outliers	0	0
Clashscore	5.73	3.68
Rotamer outliers	2.86	0.74
Average B-factors	19.01	27.29
Protein	16.16	24.32
Ligand	17.72	25.75
Solvent	34.99	46.46

*Values in parentheses are for highest-resolution shell.

Table S2: Sample preparation for Mössbauer spectroscopy

Sample	1 mM protein [µL]	1M dithionite [µL]	5M ligand [µL]
Mb* Fe(III)	500	—	—
Mb* Fe(II)	500	40	—
Mb* Fe(II) + azide	500	40	40 (Az-4)
Mb* Fe(II) + amine	500	40	40 (glycine ethyl ester)
Mb*(NMH) Fe(III)	500	—	—
Mb*(NMH) Fe(III) + EDA	500	—	40 (EDA)

Table S3: Detailed overview of the computational protocols under consideration. XC = exchange–correlation.

Parameter	Structure optimization: Protocol a	Structure optimization: Protocol b	Property calculation: Protocol α	Property calculation: Protocol β
Code + version	Gaussian 16 C.01 [7]	ORCA 4.2.1[8,9]	ORCA 4.2.1[8,9]	ORCA 4.2.1[8,9]
XC functional	PBE[10]	TPSS[11]	PBE0[12]	TPSSh[13]
Basis set H, C	def2-SVP[14]	def2-SVP[14]	def2-SVP[14]	def2-TZVP[14]
Basis set N, O	def2-SVP[14]	def2-TZVP[14]	def2-SVP[14]	def2-TZVP[14]
Basis set Fe	def2-SVP[14]	CP(PPP)[15]	def2-SVP[14]	CP(PPP)[15]
Density fitting	W06[16]	RIJK def2/JK[16]	SPLIT-RI-J def2/J[16]	RIJCOSX def2/J[16]
Dispersion correction	N/A	N/A	D3(BJ)[17,18]	D3(BJ)[17,18]
Solvation	N/A	SMD(water)[19]	CPCM(ε=80)[20]	SMD(water)[19]
Integration grid	default	2, 7 for Fe	6	6, 7 for Fe

Table S4: Coefficients and associated uncertainties (95% confidence) of the calibration functions under consideration. Curly brackets, {·}, mean "value of". In the case of protocol α, it is necessary to provide the absolute value of Δ_{QC}. The resulting prediction represents, in turn, the absolute value of Δ_{cal}. See Table C7 for raw data.

	**Isomer shift**, δ_{cal}		**Quadrupole splitting**, Δ_{cal}	
**Protocol** (property calculation)	α	β	α	β
**Intercept** / mm s^{-1}	$\{d_0\}$ = 4077.581980 ± 348.736988	$\{d_0\}$ = 6290.502352 ± 497.560851	$\{D_0\}$ = 0.1112 ± 0.1729	$\{D_0\}$ = −0.0919 ± 0.1415
**Slope** / mm s^{-1} $bohr^3$	$\{d_1\}$ = −0.3449763634 ± 0.0295058133	$\{d_1\}$ = −0.5321461394 ± 0.0420939281	$\{D_1\}$ = 0.8652 ± 0.1262	$\{D_1\}$ = 1.0087 ± 0.0706

Table S5: Experimental and predicted Mössbauer parameters of the reference systems **0a**, **0b**, and **8**. Green (no) highlighting means that the experimental isomer shift δ and quadrupole splitting Δ is within (lies outside) the 95% confidence interval of prediction, respectively. This color scheme does not apply to the asymmetry parameter η due to the lack of uncertainty quantification results for this quantity.

**Structure** (protocol)	**Protocol** α			**Protocol** β		
	δ mm s^{-1}	Δ mm s^{-1}	η	δ mm s^{-1}	Δ mm s^{-1}	η
**0a** Exp.	**0.48**	**0.95**	**N/A**	**0.48**	**0.95**	**N/A**
**0a** (a)	0.56(12)	0.94(76)	0.055	0.61(13)	1.09(70)	0.022
**0a** (b)	0.56(12)	0.97(76)	0.066	0.60(13)	1.13(70)	0.030
**0b** Exp.	**0.91**	**2.22**	**N/A**	**0.91**	**2.22**	**N/A**
**0b** (a)	0.89(13)	4.60(92)	0.004	0.58(13)	2.16(71)	0.003
**0b** (b)	0.37(11)	2.43(77)	0.055	0.42(13)	2.38(72)	0.060
**8** Exp.	**0.50**	**1.14**	**0**	**0.50**	**1.14**	**0**
**8** (a)	0.52(12)	0.56(76)	0.110	0.57(13)	0.63(70)	0.082
**8** (b)	0.51(12)	0.55(76)	0.119	0.55(13)	0.61(70)	0.083

Table S6: Binary comparison of computational protocols applied to the reference systems. "TRUE" means that the experimental value is within the 95% confidence interval of prediction, "FALSE" means that it lies outside.

**Structure**	**Protocol** (property calculation) → / **Protocol** (structure optimization) ↓	α **Isomer shift**, δ	α **Quadrupole splitting**, Δ	β **Isomer shift**, δ	β **Quadrupole splitting**, Δ
**0a**	a	TRUE	TRUE	TRUE	TRUE
**0b**	a	TRUE	FALSE	FALSE	TRUE
**8**	a	TRUE	TRUE	TRUE	TRUE
**0a**	b	TRUE	TRUE	TRUE	TRUE
**0b**	b	FALSE	TRUE	FALSE	TRUE
**8**	b	TRUE	TRUE	TRUE	TRUE

Table S7: Experimental and predicted Mössbauer parameters of the candidate systems **1**-**3**, **4a**, **4b**, and **5**-**7**. The predicted Mössbauer parameters correspond to the structures optimized with protocol a. The same color scheme as in Table C5 was applied. The color scheme of the axial Fe–N(nitrenoid) bond length, $d_{\mathrm{Fe-N}}$, refers to the uncertainty of the X-ray structure (22.9 pm, 95% confidence). The asymmetry parameter η resulting from protocol β was considered as this quantity is related to the quadrupole splitting.

**Structure**	**Protocol →** (property calculation)		α	β		
	OS[a]	$2S+1$	δ mm s^{-1}	Δ mm s^{-1}	η	$d_{\mathrm{Fe-N}}$ pm
Exp.	**II (III)**[b]	**1**	**0.38**	**−0.89**	**0.94**	**197.1**
**1**	III	2	0.24(11)	−1.86(70)	0.31	175.4
**2**	II	1	0.44(11)	−0.58(70)	0.22	186.3
**3**	II	1	0.42(11)	0.77(70)	0.08	185.2
**4a**	II	1	0.17(11)	−2.30(71)	0.64	171.3
**4b**	III	1[c]	0.20(11)	−2.20(71)	0.70	171.9
**5**	II	2	0.38(11)	−0.50(70)	0.27	180.2
**6**	II	2	0.29(11)	−1.26(70)	0.40	182.8
**7**	II	1	0.46(11)	0.30(70)	0.58	193.1

[a]Formal **o**xidation **s**tate of the iron center.
[b]An iron-III species is possible if it represents an open-shell singlet state, as is the case for system **4b**.
[c]Open-shell singlet solution.

Table S8: Results of broken-symmetry calculations. The $\langle S^2 \rangle$ expectation value and local spin densities on iron and nitrogen, $\rho(\mathrm{Fe})$ and $\rho(\mathrm{N})$, are reported in addition to the Mössbauer parameters.

**4b**	δ_{cal} mm s^{-1}	Δ_{cal} mm s^{-1}	η	$\langle S^2 \rangle$	$\rho(\mathrm{Fe})$	$\rho(\mathrm{N})$
**Reference**	**0.38**	**−0.89**	**0.94**	**1**	**±1**	**∓1**
α, `brokensym`	0.20(11)	1.87(77)	0.648	0.996	−0.838	0.728
α, `flipspin`	0.21(11)	2.03(77)	0.802	0.646	0.891	−0.751
β, `brokensym`	N/A	N/A	N/A	N/A	N/A	N/A
β, `flipspin`	0.21(13)	−2.20(71)	0.701	0.443	0.708	−0.553

Synthetic procedures

Ethyl 2-azidoacetate (Az-1)

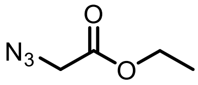

To a solution of sodium azide (270 mg, 4.15 mmol, 2.00 eq) in H_2O/acetone (1:3, 8 mL) ethyl bromoacetate (346 mg, 2.1 mmol, 1.00 eq) was added. The mixture was stirred for 30 min, diluted with DCM and washed with water. The organic layer was dried and evaporated. **Az-4** was afforded as a colorless oil (185 mg, 1.4 mmol, 70%). NMR spectra compare well with literature values.[21]

^{1}H NMR: ($CDCl_3$, 400 MHz) δ 4.26 (q, *J* = 7.1 Hz, 2H), 3.86 (s, 2H), 1.31 (t, *J* = 7.1 Hz, 3H); ^{13}C NMR: ($CDCl_3$, 400 MHz) δ 168.4, 62.0, 50.5, 14.3; HR-MS: (EI) $[M]^+$ calculated: 129.053, found 129.053.

1-Azido-butan-2-one (Az-2)

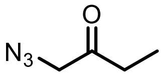

To a vigorously stirred solution of sodium azide (1.3 g, 19.8 mmol, 3.00 eq) in acetone (20 mL) 1-Bromo-2-butanone (1 g, 6.6 mmol, 1.00 eq) was added dropwise at 0°C. The solution was allowed to warm to room temperature and was stirred for 48 h. The reaction mixture was filtered over celite and concentrated in vacuo to afford the pure product as a yellow oil (640 mg, 5.66 mmol, 86%).

^{1}H NMR: ($CDCl_3$, 400 MHz) δ 3.95 (s, 2H), 2.48 (q, *J* = 7.3 Hz, 2H), 1.12 (t, *J* = 7.3 Hz, 3H); ^{13}C NMR: ($CDCl_3$, 400 MHz) δ 205.1, 57.3, 33.5, 7.5.

Ethyl-2-azidoacetamide (Az-3)

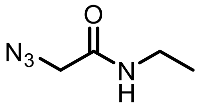

To a stirred solution of 2-chloro-N-ethylacetamide (1 g, 8.2 mmol, 1 eq) in MeCN (40 mL) was added sodium azide (1.400 g, 21.4 mmol, 2.6 eq) at room temperature. The reaction was stirred at 80 °C for 48 h. After cooling to r.t., the reaction was filtered through Celite and the solvent was removed. The product was afforded as a yellow oil (1.00 g, 7.8 mmol, 95%).

^{1}H NMR: ($CDCl_3$, 400 MHz) δ 6.29 (br, 1H), 3.98 (s, 2H), 3.33 (m, 2H), 1.17 (t, *J* = 7.3 Hz, 3H); ^{13}C NMR: ($CDCl_3$, 400 MHz) δ 166.5, 52.9, 34.5, 14.8; HR-MS: (EI) $[M]^+$ calculated: 128.069, found 129.069;

Ethyl 4-ethyl-2-methyl-1H-pyrrole-3-carboxylate

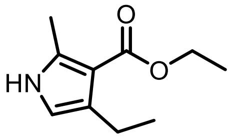

The title compound was synthesized by a two-step route following known literature procedures.[22,23]

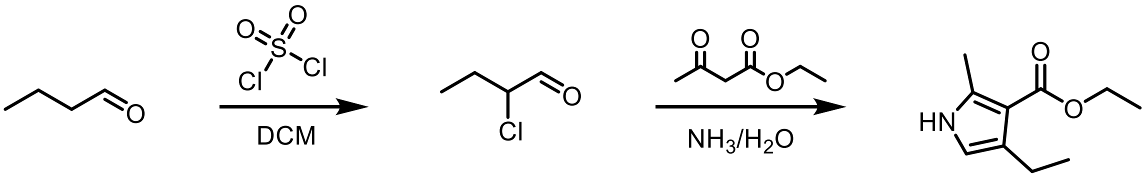

In an oven dried flask equipped with a stir bar 20 mL (16 g, 0.222 mole, 1.00 eq) butanal in 10 mL DCM were stirred at 0 °C. Slowly, 21 mL of sulfuryl chloride (35 g, 0.26 mole, 1.17 eq) were added and the mixture was stirred for 40 min at 0 °C. Then, the mixture was refluxed for another 30 min. The crude was used directly for the next step without further purification. To aqueous ammonia (50 mL 25% ammonia soloution, 50 mL water) were added 10.6 g (0.1 mole, 1.00 eq) of crude α-chlorobutyraldehyde and 13 mL (0.1 mole, 1.00 eq) ethyl acetoacetate. The mixture was stirred overnight. The next day the product was extracted with ether, washed with 10% NaOH, water, 5% HCl and then water again. The extract was dried over Na_2SO_4 and concentrated in vacuo. The product crystallized from the residues after a few hours at room temperature. Recrystallization from ether yielded the pure product as white crystals (7.91 g, 43 mmol, 19% over two steps).

^{1}H NMR ($CDCl_3$, 400 MHz) δ 7.93 (br, 1H), 6.36-6.37 (m, 1H), 4.27 (q, *J* = 7.1 Hz, 2H), 2.71 (qd, *J* = 7.4 Hz, 0.9 Hz, 2H), 2.49 (s, 3H), 1.35 (t, *J* = 7.1 Hz, 3H), 1.18 (t, *J* = 7.4 Hz, 3H) ; ^{13}C NMR ($CDCl_3$, 400 MHz) δ 166.1, 136.0, 128.7, 112.9, 110.2, 77.3, 76.7, 59.1, 20.2, 14.6, 14.5, 14.2; HR-MS (ESI) $[M+Na]^+$ calculated: 204.099, found: 204.099;

Preparation of ^{57}Fe heme

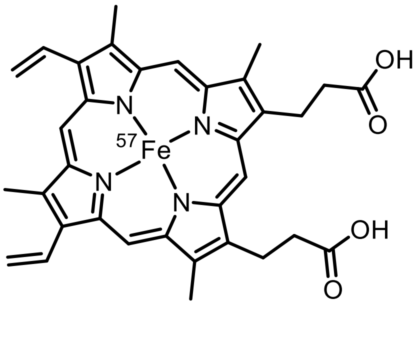

To synthesize iron chloride, 50 mg of iron powder (0.896 mmol) were dissolved and stirred in 2 mL concentrated hydrochloric acid until all iron was dissolved. The solvent was evaporated and the product was obtained as a yellow solid. Separate analysis of the product with potassium ferrocyanide and potassium ferricyanide resulted in formation of blue pigments, suggesting the presence of Fe^{3+} and Fe^{2+} ions. The mixture was used for further steps without further purification. 50 mg (0.089 mmol) protoporphyrin IX was dissolved in 5 mL acetic acid and a few drops of HCl were added. 20 mg of the iron salt was dissolved in 0.5 mL 90 % acetic acid. The two solutions were combined and then filtered. The solution was heated to reflux and refluxed until no porphyrin fluorescence was detected. Then solid sodium acetate was added and heating was stopped. The crystallization process was allowed to occur overnight. The next day, crystals were recovered by centrifugation, washed with concentrated acetic acid, diluted acetic acid, water, diethyl ether and subsequently dried in the oven at 120 °C for 2 h. The product was obtained as a black solid (31 mg, 0.048 mmol, 55%). Analysis of the product was carried out by LC-MS by comparing retention times and masses of heme and the product.

COMPUTATIONAL DETAILS

Quantum chemical calculations

Structure models

Schematic structures of the species under consideration (**0a**, **0b**, **1**-**3**, **4a**, **4b**, **5**-**8**) are shown in Figures S8 and S9. Crystal structures of the resting state (**0a**, **0b**), the amine (**8**), and the candidate systems (**1**-**3**, **4a**, **4b**, **5**-**7**) were extracted from PDB files. The protein surroundings were removed, except for the histidine axial ligand of the iron complex, which was truncated to an imidazole ligand. All structures were further simplified: porphyrin substituents were replaced with hydrogen nuclei and the ester group of the axial ligand (opposite to the imidazole ligand) was replaced with the free acid (does not apply to the resting states **0a** and **0b**).

Computational protocols

We developed computational protocols for both structure optimization (protocols `a` and `b`) and subsequent property calculations (protocol α and β), see Table S3. Protocols `a` and α were developed on the basis of previous work by Proppe and Reiher on theoretical Mössbauer spectroscopy[24] and then applied to heme complexes.[1] Protocols `b` and β were taken without change from work by Krewald and colleagues.[25]

Following experimental findings, all systems were considered in low-spin states except for the resting state **0b** (high-spin state). All optimized structures were confirmed as local minima by frequency calculations.

The Karlsruhe def2 family of basis sets[14] was used in all calculations and for all elements except for iron, for which Neese's adapted basis for core densities, CP(PPP),[15] was chosen. For all property calculations, the grid accuracy parameter for iron was increased to 7 in ORCA's unit system.

Broken-symmetry calculations

The structure optimization of the open-shell singlet species **4b** was based on protocol `a` using the guess=mix keyword in Gaussian 16 to enhance convergence to a broken-symmetry solution. In subsequent property calculations (ORCA), broken-symmetry solutions were searched for by either using the `brokensym` keyword or by manually inverting the spin on a specific atom (`flipspin` keyword).

Electronic hyperfine interactions

Each property calculation (resulting from either protocol α or protocol β) yields three quantities associated with the electronic hyperfine interactions probed by Mössbauer spectroscopy: the contact

density $\rho(\mathbf{R}_{\mathrm{Fe}})$ at the Fe nucleus ($\mathbf{R}_{\mathrm{Fe}}$ denotes the Cartesian coordinates of the nuclear center), the uncalibrated quadrupole splitting Δ_{QC}, and the asymmetry parameter η. The first two quantities require calibration to ensure comparability against experimental data (see below).

Mössbauer parameter calibration

Both $\rho(\mathbf{R}_{\mathrm{Fe}})$ and Δ_{QC} enter linear calibration functions to obtain predictions of the isomer shift, δ_{cal},

$$\delta_{\mathrm{cal}} = d_0 + d_1 \rho(\mathbf{R}_{\mathrm{Fe}})$$

and the quadrupole splitting, Δ_{cal},

$$\Delta_{\mathrm{cal}} = D_0 + D_1 \Delta_{\mathrm{QC}}$$

The values of the coefficients d_0, d_1, D_0, and D_1 were obtained by fitting $\rho(\mathbf{R}_{\mathrm{Fe}})$ and Δ_{QC} to experimental data (δ_{exp} and Δ_{exp}, respectively) via the aforementioned calibration functions (see appendix for raw data). Note that there is no theoretical justification for the coefficient D_0 and, hence, its value should be monitored carefully.

In previous work,[24,25] we showed that the standard deviation of predictions associated with both δ_{cal} and Δ_{cal} is substantially larger (>0.05 mm s^{-1} and >0.35 mm s^{-1}, respectively) than the uncertainty of measurement associated with δ_{exp} and Δ_{exp}, which can be taken as 0.02 mm s^{-1} and 0.03 mm s^{-1} at 80 K,[26] respectively. Therefore, the quantification of prediction uncertainty is an essential computational step that should not be neglected in the analysis and evaluation of theoretical Mössbauer parameters.

Both parameter fitting and uncertainty quantification were conducted in parallel by bootstrapping an ensemble of linear least-square regression models. The ensemble was created by drawing samples from a known distribution of N data points: the data set at hand. The various bootstrapping variants[27,28] have in common that they assign each data point a random weight p_i in each sample, which can be interpreted as a probability because the weights add up to unity, $\sum_{i=1}^{N} p_i = 1$.

The following procedure describes how these weights were obtained by sampling from a uniform Dirichlet distribution,[29] which is known as Bayesian bootstrapping.[30] To a tuple of $N-1$ uniformly sampled random numbers in the range $[0, 1]$, the numbers 0 and 1 are appended. The tuple is then

sorted in ascending order, yielding $q_0 = 0 < q_1 < \cdots < q_{N-1} < q_N = 1$. The weight of the ith data point is obtained by subtracting the $(i-1)$-th from the ith random number,

$$p_i = q_i - q_{i-1}$$

This resulting weighted data set is called a bootstrap sample and the sampling process is repeated for a total of B times. Throughout this work, $B = 10{,}000$ samples were drawn.

Each sample yields a unique set of values of the coefficients d_0, d_1, D_0, and D_1. Hence, for each coefficient, we obtained a distribution of 10,000 values. Propagating these distributions through the calibration functions, we obtain distributions of δ_{cal} and Δ_{cal}, respectively. By assuming normally distributed Mössbauer parameters, multiplying the standard deviation of their distributions with a factor of 1.96 yields a 95% confidence interval, which we define here as prediction uncertainty.

As the protocols α and β use different computational settings, they require individual calibration. The results are summarized in Table S4 (see Appendix for raw data). The Mössbauer parameters associated with protocol β were obtained from a Jupyter notebook that is openly accessible at https://git.rwth-aachen.de/krewald/mossbauerprediction. Details on its development can be found in Ref. C3. This notebook served as a basis for predicting Mössbauer parameters associated with protocol α. It differs from the original notebook in the experimental training data used in the calibration procedure. Here, the systems 1-44 of Ref. C1 were considered except for complexes 3, 7, 13, 14, 15, 17, 20, 22, 24, 26, 27, 28, 30, 36. We discarded these systems for the sake of consistency. The remaining 30 systems represent mononuclear complexes without significant spin contamination. Furthermore, we considered only absolute values of the quadrupole splitting in the case of protocol α as the sign of Δ_{exp} has not been consistently reported. Both calibration notebooks are included in an openly accessible repository associated with this Supporting Information (see the publisher's webpage).

COMPUTATIONAL RESULTS

Contact densities, $\rho(\mathbf{R}_{\mathrm{Fe}})$, and uncalibrated quadrupole splittings, Δ_{QC}, can be found in the subsection "Raw data" at the end of this section.

Comparison of protocols

Calculations using all combinations of the four protocols a, b, α, β (α/a, β/a, α/b, β/b) were performed on the reference structures of the resting states (**0a**, **0b**) and the amine (**8**), with the goal

of determining which combinations reproduce the experimental Mössbauer parameters for these systems. The results are shown in Table S5.

To better distinguish between quantitative and qualitative disagreement of these results, a binary comparison is shown in Table S6. For each combination of i) Mössbauer parameter (δ, Δ), ii) reference system (**0a**, **0b**, **8**), iii) structure optimization protocol (a, b), and iv) property calculation protocol (α, β), either of the two possible labels "TRUE" and "FALSE" was assigned. If "TRUE", the corresponding experimental value is within the 95% confidence interval of prediction. Otherwise, the label is "FALSE". It should also be noted that the prediction uncertainty of the quadrupole splitting is exceptionally large (>0.70 mm s^{-1} for both α and β). Of course, an experimental value lying outside the confidence interval of prediction is not necessarily "wrong"; there is a 5% chance that it is valid (assuming the confidence interval is valid itself). Hence, there is a risk of 5% that our decision boundary is misleading.

While protocol α/a is able to reproduce the experimental isomer shift δ_{exp} of all reference systems, it fails to reproduce the experimental quadrupole splitting Δ_{exp} of the high-spin resting state **0b**. The opposite case is observed for protocol β/a. It reproduces Δ_{exp} of all reference systems but fails to reproduce δ_{exp} of **0b**. In fact, there is not a single protocol that can reproduce both Mössbauer parameters of system **0b**. Both protocols α/b and β/b reproduce Δ_{exp} of all reference systems, but neither of them is able to reproduce δ_{exp} of system **0b**. Given that only protocol a (structure optimization) could reproduce all experimental values of both isomer shift (in combination with protocol α) and quadrupole splitting (in combination with protocol β), we decided to discard the structure optimization protocol b for the systems investigated in this study. Following the same line of argument, we also decided to apply different protocols for the calculation of the contact density $\rho(\mathbf{R}_{\mathrm{Fe}})$ (α) and the uncalibrated quadrupole splitting Δ_{QC} (β). We rationalize this decision by the fact that approximations inherent to the exchange–correlation functional have different effects on the individual properties. This decision would not be acceptable, however, if any of the two protocols would yield inconsistent results within its associated property, which is not the case here.

Analysis of candidate systems

Structure optimizations and property calculations were carried out for all candidate systems (**1**-**3**, **4a**, **4b**, **5**-**7**) with the protocols selected in the previous subsection (α/a and β/a). The results are shown in Table S7.

In the case of protocol α, the Mössbauer parameters of the open-shell singlet state **4b** represent the average of both broken-symmetry results (`brokensym` and `flipspin`), whereas it represents the `flipspin` result only in the case of protocol β, see the next subsection for more details. In addition to the Mössbauer parameters, we provided the Fe–N(candidate) bond length, $d_{\mathrm{Fe-N}}$, for each system. Given that uncertainty information is not available for the asymmetry parameter η combined with the finding that none of its calculated values appears close to the experimental reference value, we neglect this quantity in the following discussion.

Considering the experimental Mössbauer and structural parameters provided in Table S7, we propose to discard systems **1**, **4a**, and **4b**. In addition, we propose to discard systems **5** and **6** as they do not represent the experimentally determined spin state. Eventually, we consider systems **2**, **3**, and **7** possible candidates. Only these systems match with all experimental constraints considered in this study despite the low resolution of DFT calculations.

<u>Results of broken-symmetry calculations</u>

The molecular structure of the open-shell singlet state **4b** obtained via protocol a resembles that of the closed-shell singlet state **4a**. The results of the property calculations are shown in Table S8. The SCF procedure of the `brokensym` calculation with protocol β did not converge. The solution "α, brokensym" shows the closest resemblance with a broken-symmetry solution with respect to the $\langle S^2 \rangle$ expectation value and the spin density on iron and nitrogen, $\rho(\mathrm{Fe})$ and $\rho(\mathrm{N})$, respectively.

Appendix:

Raw data: Contact densities, $\rho(\mathbf{R}_{\text{Fe}})$, and uncalibrated quadrupole splittings, Δ_{QC}, for all combinations of computational protocols.

Structure (protocol)	Protocol α		Protocol β	
	$\rho(\mathbf{R}_{\text{Fe}})$ bohr^{-3}	Δ_{QC} mm s^{-1}	$\rho(\mathbf{R}_{\text{Fe}})$ bohr^{-3}	Δ_{QC} mm s^{-1}
**0a** (a)	11818.261168242	0.957	11819.866515133	1.173
**0a** (b)	11818.277154563	0.991	11819.876119818	1.212
**0b** (a)	11817.303803640	5.184	11819.913176931	2.237
**0b** (b)	11818.827897139	2.685	11820.224118530	2.453
**1** (a)	11819.206452699	−2.218	11820.506816782	−1.755
**1** (b)	11819.236293129	−1.889	11820.529312212	−1.479
**2** (a)	11818.620552240	−0.371	11820.131408336	−0.483
**2** (b)	11818.674382977	0.398	11820.167013533	−0.429
**3** (a)	11818.683864126	−0.533	11820.166305114	−0.669
**3** (b)	11818.732611119	−0.543	11820.205068312	−0.668
**4a** (a)	11819.401905361	−2.270	11820.673327682	−2.159
**4a** (b)	11819.424585949	−2.243	11820.678211257	−2.120
**4b** (a) `brokensym`	11819.306074658	−2.028	N/A	N/A
**4b** (a) `flipspin`	11819.280821771	−2.214	11820.617836301	−2.086
**5** (a)	11818.802398287	0.735	11820.265919157	−0.404
**5** (b)	11818.774188152	0.762	11820.196105435	−0.487
**6** (a)	11819.047867902	−1.421	11820.356675572	−1.159
**6** (b)	11819.072596481	−1.427	11820.373226611	−1.154
**7** (a)	11818.570297335	0.170	11820.081263376	0.392
**7** (b)	11818.554476486	0.222	11820.064100585	0.445
**8** (a)	11818.378698468	0.521	11819.940174789	0.711
**8** (b)	11818.410846302	0.508	11819.963955260	0.695

BIBLIOGRAPHY:

(1) Hayashi, T.; Tinzl, M.; Mori, T.; Krengel, U.; Proppe, J.; Soetbeer, J.; Klose, D.; Jeschke, G.; Reiher, M.; Hilvert, D. Capture and Characterization of a Reactive Haem–Carbenoid Complex in an Artificial Metalloenzyme. *Nat. Catal.* **2018**, *1*, 578–584.

(2) Green, A. P.; Hayashi, T.; Mittl, P. R. E.; Hilvert, D. A Chemically Programmed Proximal Ligand Enhances the Catalytic Properties of a Heme Enzyme. *J. Am. Chem. Soc.* **2016**, *138*, 11344–11352.

(3) Adams, P. D.; Afonine, P. V.; Bunkóczi, G.; Chen, V. B.; Davis, I. W.; Echols, N.; Headd, J. J.; Hung, L. W.; Kapral, G. J.; Grosse-Kunstleve, R. W.; McCoy, A. J.; Moriarty, N. W.; Oeffner, R.; Read, R. J.; Richardson, D. C.; Richardson, J. S.; Terwilliger, T. C.; Zwart, P. H. PHENIX: A Comprehensive Python-Based System for Macromolecular Structure Solution. *Acta Crystallogr. Sect. D Biol. Crystallogr.* **2010**, *66*, 213–221.

(4) Emsley, P.; Cowtan, K. Coot: Model-Building Tools for Molecular Graphics. *Acta Crystallogr. Sect. D Biol. Crystallogr.* **2004**, *60*, 2126–2132.

(5) Parak, F.; Prusakov, V. E. Relaxation of Non-Equilibrium States of Myoglobin Studied by Mössbauer Spectroscopy. *Hyperfine Interact.* **1994**, *91*, 885–890.

(6) Prusakov, V. E.; Steyer, J.; Parak, F. G. Mössbauer Spectroscopy on Nonequilibrium States of Myoglobin: A Study of r-t Relaxation. *Biophys. J.* **1995**, *68*, 2524–2530.

(7) Frisch, M. J.; Trucks, G. W.; Schlegel, H. B.; Scuseria, G. E.; Robb, M. a.; Cheeseman, J. R.; Scalmani, G.; Barone, V.; Petersson, G. a.; Nakatsuji, H.; Li, X.; Caricato, M.; Marenich, a. V.; Bloino, J.; Janesko, B. G.; Gomperts, R.; Mennucci, B.; Hratchian, H. P.; Ortiz, J. V.; Izmaylov, a. F.; Sonnenberg, J. L.; Williams; Ding, F.; Lipparini, F.; Egidi, F.; Goings, J.; Peng, B.; Petrone, A.; Henderson, T.; Ranasinghe, D.; Zakrzewski, V. G.; Gao, J.; Rega, N.; Zheng, G.; Liang, W.; Hada, M.; Ehara, M.; Toyota, K.; Fukuda, R.; Hasegawa, J.; Ishida, M.; Nakajima, T.; Honda, Y.; Kitao, O.; Nakai, H.; Vreven, T.; Throssell, K.; Montgomery Jr., J. a.; Peralta, J. E.; Ogliaro, F.; Bearpark, M. J.; Heyd, J. J.; Brothers, E. N.; Kudin, K. N.; Staroverov, V. N.; Keith, T. a.; Kobayashi, R.; Normand, J.; Raghavachari, K.; Rendell, a. P.; Burant, J. C.; Iyengar, S. S.; Tomasi, J.; Cossi, M.; Millam, J. M.; Klene, M.; Adamo, C.; Cammi, R.; Ochterski, J. W.; Martin, R. L.; Morokuma, K.; Farkas, O.; Foresman, J. B.; Fox, D. J. G16_C01. 2016, p Gaussian 16, Revision C.01, Gaussian, Inc., Wallin.

(8) Neese, F. The ORCA Program System. *WIREs Comput. Mol. Sci.* **2012**, *2*, 73–78.

(9) Neese, F. Software Update: The ORCA Program System, Version 4.0. *WIREs Comput. Mol. Sci.* **2018**, *8*, e1327.

(10) Perdew, J. P.; Burke, K.; Ernzerhof, M. Generalized Gradient Approximation Made Simple. *Phys.*

Rev. Lett. **1996**, *77*, 3865–3868.

(11) Tao, J.; Perdew, J. P.; Staroverov, V. N.; Scuseria, G. E. Climbing the Density Functional Ladder: Nonempirical Meta--Generalized Gradient Approximation Designed for Molecules and Solids. *Phys. Rev. Lett.* **2003**, *91*, 146401.

(12) Adamo, C.; Barone, V. Toward Reliable Density Functional Methods without Adjustable Parameters: The PBE0 Model. *J. Chem. Phys.* **1999**, *110*, 6158–6170.

(13) Staroverov, V. N.; Scuseria, G. E.; Tao, J.; Perdew, J. P. Comparative Assessment of a New Nonempirical Density Functional: Molecules and Hydrogen-Bonded Complexes. *J. Chem. Phys.* **2003**, *119*, 12129–12137.

(14) Weigend, F.; Ahlrichs, R. Balanced Basis Sets of Split Valence, Triple Zeta Valence and Quadruple Zeta Valence Quality for H to Rn: Design and Assessment of Accuracy. *Phys. Chem. Chem. Phys.* **2005**, *7*, 3297–3305.

(15) Neese, F. Prediction and Interpretation of the 57Fe Isomer Shift in Mössbauer Spectra by Density Functional Theory. *Inorganica Chim. Acta* **2002**, *337*, 181–192.

(16) Weigend, F. Accurate Coulomb-Fitting Basis Sets for H to Rn. *Phys. Chem. Chem. Phys.* **2006**, *8*, 1057–1065.

(17) Grimme, S.; Antony, J.; Ehrlich, S.; Krieg, H. A Consistent and Accurate *ab initio* Parametrization of Density Functional Dispersion Correction (DFT-D) for the 94 Elements H-Pu. *J. Chem. Phys.* **2010**, *132*, 154104.

(18) Grimme, S.; Ehrlich, S.; Goerigk, L. Effect of the Damping Function in Dispersion Corrected Density Functional Theory. *J. Comput. Chem.* **2011**, *32*, 1456–1465.

(19) Marenich, A. V; Cramer, C. J.; Truhlar, D. G. Universal Solvation Model Based on Solute Electron Density and on a Continuum Model of the Solvent Defined by the Bulk Dielectric Constant and Atomic Surface Tensions. *J. Phys. Chem. B* **2009**, *113*, 6378–6396.

(20) Barone, V.; Cossi, M. Quantum Calculation of Molecular Energies and Energy Gradients in Solution by a Conductor Solvent Model. *J. Phys. Chem. A* **1998**, *102*, 1995–2001.

(21) Shi, F.; Waldo, J. P.; Chen, Y.; Larock, R. C. Benzyne Click Chemistry: Synthesis of Benzotriazoles from Benzynes and Azides. *Org. Lett.* **2008**, *10*, 2409–2412.

(22) Stevens, C. L.; Farkas, E.; Gillis, B. Epoxyethers. VII. Reaction of α-Haloaldehydes with Base. *J. Am. Chem. Soc.* **1954**, *76*, 2695–2698.

(23) Roomi, M. W.; MacDonald, S. F. The Hantzsch Pyrrole Synthesis. *Can. J. Chem.* **1970**, *48* , 1689–1697.

(24) Proppe, J.; Reiher, M. Reliable Estimation of Prediction Uncertainty for Physicochemical Property Models. *J. Chem. Theory Comput.* **2017**, *13*, 3297–3317.

(25) Gallenkamp, C.; Kramm, U. I.; Proppe, J.; Krewald, V. Calibration of Computational Mössbauer Spectroscopy to Unravel Active Sites in FeNC Catalysts for the Oxygen Reduction Reaction. *Int. J. Quantum Chem.* **2021**, *121*, e26394.

(26) Li, M.; Bonnet, D.; Bill, E.; Neese, F.; Weyhermüller, T.; Blum, N.; Sellmann, D.; Wieghardt, K. Tuning the Electronic Structure of Octahedral Iron Complexes [FeL(X)] (L = 1-Alkyl-4,7-Bis(4-Tert-Butyl-2-Mercaptobenzyl)-1,4,7-Triazacyclononane, X = Cl, CH3O, CN, NO). The S = 1/2 ⇌ S = 3/2 Spin Equilibrium of [FeLPr(NO)]. *Inorg. Chem.* **2002**, *41*, 3444–3456.

(27) Davison, A. C.; Hinkley, D. V. *Bootstrap Methods and Their Application*; Cambridge University Press: Cambridge, 1997.

(28) Chernick, M. R. *Bootstrap Methods: A Guide for Practictioners and Researchers*; Wiley-Interscience: Hoboken, 2008.

(29) Bishop, C. M. *Pattern Recognition and Machine Learning*; Springer: New York, 2006.

(30) Rubin, D. B. The Bayesian Bootstrap. *Ann. Stat.* **1981**, *9*, 130–134.
